# Improving reproducibility and translational potential of mouse models: lessons from studying leishmaniasis

**DOI:** 10.3389/fimmu.2025.1559907

**Published:** 2025-04-22

**Authors:** Mahmoud Nateghi-Rostami, Marie Lipoldová, Yahya Sohrabi

**Affiliations:** ^1^ Department of Parasitology, Pasteur Institute of Iran, Tehran, Iran; ^2^ Department of Medical Genetics, Third Faculty of Medicine, Charles University, Prague, Czechia; ^3^ Institute of Molecular Genetics, Czech Academy of Sciences, Prague, Czechia; ^4^ Department of Cardiology I-Coronary and Peripheral Vascular Disease, Heart Failure, University Hospital Münster, University of Münster, Münster, Germany

**Keywords:** mouse model, human leishmaniasis, translation, influencing factor, experimental analysis, reproducibility of data, experimental conditions

## Abstract

Leishmaniasis is a complex disease caused by protozoan parasites of the genus *Leishmania*, which are transmitted by phlebotomine sand flies. The clinical manifestations of leishmaniasis are diverse, ranging from self-healing cutaneous lesions to fatal systemic disease. Mouse models are instrumental in advancing our understanding of the immune system against infections, yet their limitations in translating findings to humans are increasingly highlighted. The success rate of translating data from mice to humans remains low, largely due to the complexity of diseases and the numerous factors that influence the disease outcomes. Therefore, for the effective translation of data from murine models of leishmaniasis, it is essential to align experimental conditions with those relevant to human infection. Factors such as parasite characteristics, vector-derived components, host status, and environmental conditions must be carefully considered and adapted to enhance the translational relevance of mouse data. These parameters are potentially modifiable and should be carefully integrated into the design and interpretation of experimental procedures in *Leishmania* studies. In the current paper, we review the challenges and perspective of using mouse as a model for leishmaniasis. We have particularly emphasized the non-genetic factors that influence experiments and focused on strategies to improve translational value of studies on leishmaniasis using mouse models.

## 1 Introduction

Leishmaniasis is a complex disease caused by protozoan parasites from more than 20 *Leishmania* species, which are transmitted by over 90 different species of phlebotomine sand flies (1, 2). Among over 800 species of sand flies recorded, 98 are proven or suspected vectors of human leishmaniases; these include 42 *Phlebotomus* species in the Old World and 56 *Lutzomyia* species in the New World (all: Diptera: Psychodidae) (3). The inoculated parasites infect the so-called professional phagocytes (neutrophils, monocytes, and macrophages), as well as dendritic cells and fibroblasts (4–6). Leishmaniasis affects various mammalian hosts, offering diverse opportunities to study immunopathology, genetic control, and host-parasite interactions using animal models. The clinical manifestations of leishmaniasis differ significantly, from self-healing cutaneous lesion to severe systemic disease in humans and asymptomatic infections in many mammals. This variability presents significant challenges in selecting appropriate animal models, which must be carefully aligned with the specific objectives of the study. Various models, including mice, hamsters, dogs, and non-human primates, have been developed to investigate leishmaniasis’ pathology, disease mechanisms, and potential therapeutic or vaccine candidates (7–11). Among these, the mouse has emerged as the most prominent model due to its genetic tractability, short lifespan, and physiological similarities to humans.

Infection with *Leishmania* species manifests in a wide spectrum of clinical forms, including cutaneous leishmaniasis (CL), mucocutaneous leishmaniasis (MCL), visceral leishmaniasis (VL), and post-kala-azar dermal leishmaniasis (PKDL) types (**Table 1**). CL is the most common form of disease that typically begins with the formation of a papule at the site of a female sand fly bite, which progressively enlarges and develops into a nodule or probably to a painless open ulcer. While CL is normally a self-healing disease with spontaneous cure without any treatment, the course of infection can vary depending on the host’s immune response and the infecting *Leishmania* species. MCL arises as a metastatic complication of CL when parasites disseminate through the lymphatic system to the mucosal tissues resulting in destruction and disfigurement. VL is the most severe form of leishmaniasis associated with high fatality rate without proper treatment. Most cases of VL do not show clinical disease and remain asymptomatic, but in active VL the parasites spread to reticuloendothelial system (RES). This leads to systemic disease characterized by prolonged fever, weight loss, hepatosplenomegaly and anemia. PKDL emerges as a complication in some patients who recovered from VL in endemic areas. PKDL is characterized by macular, maculopapular, and nodular rash. PKDL patients serve as a significant reservoir for disease transmission.

**Table 1:** Main species of *Leishmania* causing human disease and their characteristics.

Subgenus	Species	Old/New World	Clinical form	Main reservoir	Geographical distribution	References
***Leishmania subgenus***	*L. donovani* *(Syn. of L. archibaldi)*	Old World	a VL and PKDL rarely CL	Humans dogs	India, Bangladesh, Ethiopia, and Sudan	(2, 191–193)
	*Leishmania tropica* *(Syn. of L. killicki)*	Old World	CL, LR, and rarely VL	Humans	Eastern Mediterranean, the Middle East, and Northeastern and Southern Africa	(2, 191–194)
	*Leishmania aethiopica*	Old World	CL, DCL, DsCL	Rock Hyraxes	Ethiopia and Kenya	(2, 191–193, 195)
	*Leishmania major*	Old World	CL	Rodents	north Africa, the Middle East, Central Asia, and West Africa	(2, 191–194)
	*Leishmania infantum* *(Syn. of L. chagasi)*	Old & New Worlds	VL and sometimes CL	Humans (Dogs, Cats, Foxes, Jackals)	China, Southern Europe, Transcaucasia, South America, Mediterranean basin, Asia, Latin America	(2, 191–193, 196)
	*Leishmania mexicana* *(Syn. Of L. pifanoi)*	New World	CL, DCL, and DsCL	Forest Rodents and marsupials	Central and South America	(2, 191–193, 197)
	*Leishmania amazonensis* *(Syn. of L. garnhami)*	New World	CL, DCL, and DsCL	Possums and rodents	South America	(2, 191–193, 197)
***Viannia subgenus***	*Leishmania braziliensis*	New World	CL, MCL, DCL, and LR	Dogs, humans, rodents, and horses, Sloth	Central and South America	(2, 191–193, 198)
	*Leishmania guyanensis*	New World	CL, DsCL, and MCL	Possums, sloths, and anteaters	South America	(2, 191–193, 198, 199)
	*Leishmania panamensis*	New World	CL, MCL	Sloth	Central and South America	(2, 191–193, 198)

VL, visceral leishmaniasis; PKDL, post-kala-azar dermal leishmaniasis; CL, cutaneous leishmaniasis; LR, leishmaniasis recidivans; DCL, diffuse cutaneous leishmaniasis; DsCL, disseminated cutaneous leishmaniasis; MCL, mucocutaneous leishmaniasis. Bold letters highlight the two main Leishmania species responsible for causing CL.

It is important to note that the majority of *Leishmania* infections in humans, particularly in VL cases, remain asymptomatic (12, 13). In endemic regions, most individuals exposed to the parasite are able to control the infection through induction of a robust protective immune response and development of immunological memory (4, 14). Studies in both mice and humans indicate that the outcome of *Leishmania* infection is influenced by a complex interplay between host factors, parasite-specific characteristics and environmental conditions (15–18). Mice and human share more than 90% of their genome. Mice are easy to breed and maintain, they are useful tools for genetic manipulation and conditional experiments that are usually not possible in human (19). However, despite considerable similarities and numerous advantages, significant differences in their physiology and genetics also exist. These differences, together with environmental factors, influence potential of mouse models to accurately mimic human diseases. For example, mice have a shorter life span and different physiological characteristics, such as hearth beat, body temperature, active/sleeping time, diet, and microbiota composition. In addition, mice are used as a model for some diseases that naturally do not occur in mice such as leishmaniasis. There is a significant overlap in the clinical manifestation of leishmaniasis between mice and human, however, mice and human also exhibit considerable differences in developing the symptoms. For instance, infection with *L. major* usually does not visceralize in human, while parasite disseminates to visceral organs in susceptible mice. Furthermore, in order to increase the translational capacity of mouse data, influencing parameters should be well characterized, carefully adapted, and considered when designing an experiment and interpreting results in *Leishmania* studies. Animal models including mouse, golden hamster, dog and monkey have been used *in vivo* testing of new antileishmanial agent (20–27). To evaluate drug efficacy, choosing an animal with closer evolutionary relatedness might be better. On the other side, mouse model provides fast answer to evaluate some parameters such as toxicity and dose response. According to recent data mouse models are by far the most commonly used animal model for antileishmanial drug discovery (27, 28). Several compounds such as miltefosine, amphotericin B etc. have been tested in mouse models (28–31). Depending on drug formulation, administration route and treatment protocol varies from topical, oral administration or inoculation (30–33). Due to the distinct phylogeny and differences between human and mice, the data may not be predictive of the response in human because compounds may show an effective response in animal model, but has no or very low efficacy in human (34). In addition, in human treatment starts when the clinical symptoms appears, whereas, in mouse animal model particular in mice, treatment usually starts only week or weeks after parasite challenge, which might result in different result than in human (10). The current paper provides comprehensive information of non-genetic influencing factors that limit the translational value of mouse models and offers strategies to improve their relevance to human leishmaniasis.

## 2 Host genetics

Leishmaniasis is a complex disease with pathogenesis influenced by various factors, including environmental conditions, insect vector, and genetic makeup of both the parasite and the host. Host genetics is especially intriguing because clinical outcomes can differ greatly among patients infected with the same *Leishmania* species and sharing similar non-genetic factors (35).

Mice are the most widely used model in identifying genetic control of leishmaniasis. The genetic control of susceptibility to various *Leishmania* species in mouse models has been extensively studied, with several loci linked to disease outcomes. Interestingly, many of the susceptibility genes or loci identified in mice overlap with human genes that play a role in regulating disease severity (36–48). Genome-wide linkage analysis identified more than thirty loci that control susceptibility to *Leishmania* infection in mice (9), but only two of them have been translated to human. The role of *Nramp1/Slc11a1* that is linked to leishmaniasis in mice (49) has also been proved in human (50). Wound healing related gene *Fli1* that influences cutaneous leishmaniasis caused by *L. major* in mouse (51) has impact on susceptibility to *L. braziliensis* caused CL in human (52).

One of the main reasons for low degree of translational potential of mouse to human concerning genetics studies is the low polymorphic complexity of mouse genome in comparison to highly heterogenic human genome. Genetic studies have been mostly performed on a limited number of inbred strains that do not mimic the high genetic polymorphism observed in the human genome. On one hand, the lower genetic complexity of inbred strains offers an advantage to study mechanism of the diseases; but on the other hand, it fails to show the network of gene-gene interactions in the human genome that play a crucial role in the disease control. Using tools such as crossing two inbreed strains improved the efficiency of mapping of complex quantitative trait loci (QTLs) revealed the network of gene-gene interactions (9, 37, 40, 41, 43, 44, 46, 47, 48). In addition, murine models do not fully recapitulate the complexity of human disease, in part due to intricate interactions between host genetics and environmental factors. This limitation can be partially addressed by employing a broader range of mouse strains with diverse genetic backgrounds (including wild-derived strains), optimization of experimental conditions to reduce limiting factors should be considered. In contrast to mice especially SPF kept mice, human population are heavily encountered with different infections in daily basis, therefore, the genetic polymorphism associated to susceptibility to infectious diseases are under selective pressure (53). The genetic control of leishmaniasis and influence of the host genetic factors in pathogenesis of leishmaniasis has been thoroughly discussed elsewhere (under review).

## 3 Non-genetic parameters influencing *Leishmania* infection

**Figure 1:**
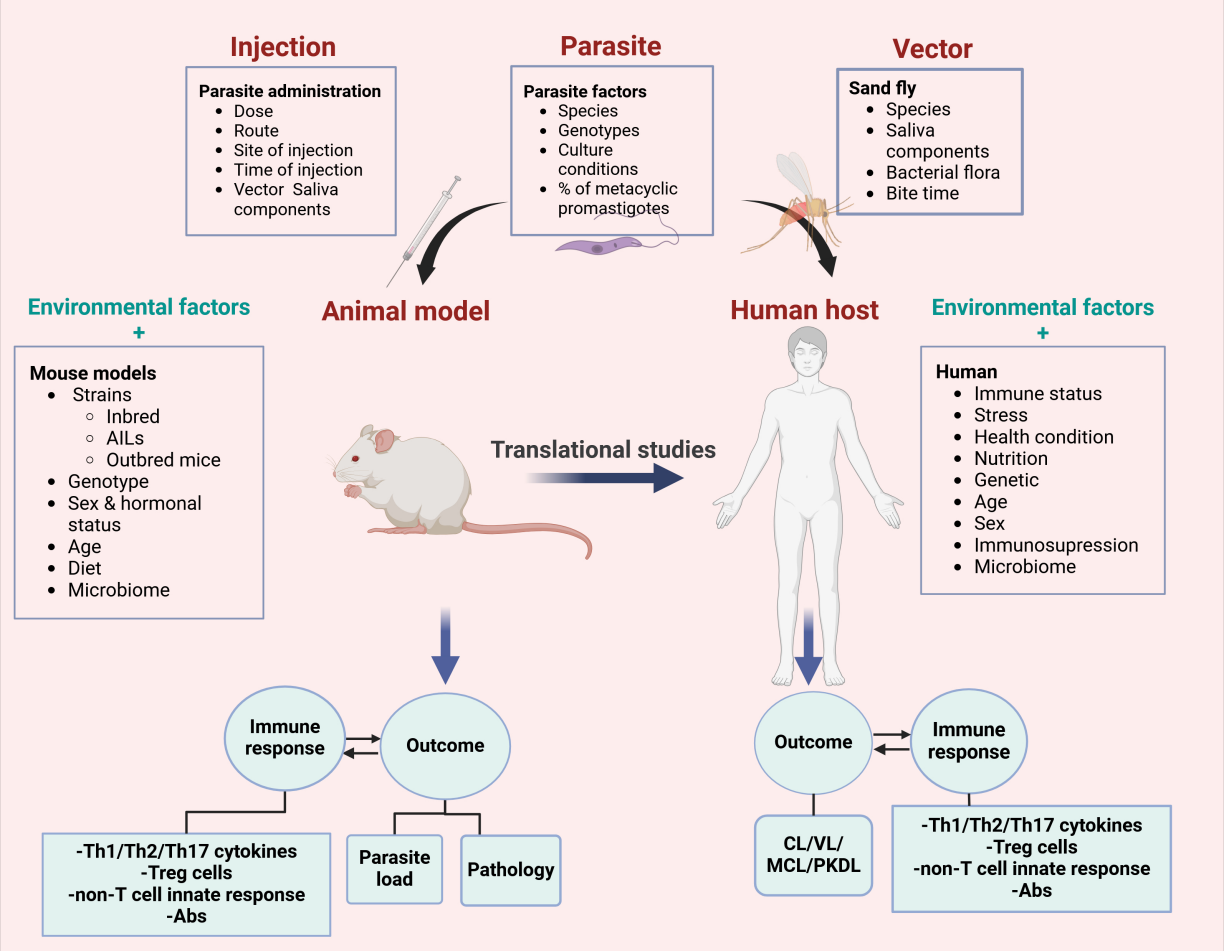
Factors that influence translational potential of mouse models to human leishmaniasis. Many parameters such as parasite, vector and host related factors might have significant impact on the disease outcome in mice and human. In addition to host factors and environment parameters, parasite, vector or inoculation can change the responses to the parasite infection. Therefore, these factors must be adapted and modified in order to increase translational value of mouse results.

A significant proportion of data on the mechanisms of parasite pathogenesis and host immune responses have been collected from animal models. Although the data generated from the experimental models are pivotal, translating the results obtained from experimental studies to human is challenging. Primary goal in developing animal models has often focused on replicating human-like phenotypes, which can differ significantly from their natural forms. Moreover, the infection in experimental models is influenced by various factors such as parasite species (2) and sub-strains (54, 55), dose (56), injection route (57), genetic background of the host (41), sex (58, 59) and hormonal status (60), age (61), microbiome composition (62), as well as presence of other infections (63). *In vitro* and *in vivo* experiments show influence of culture conditions (64) and medium composition (65) on parasite infectivity (65) and virulence capabilities (64, 66). These parameters can be controlled and should be described in experimental protocols (**Figure 1**). In addition, there are differences between infection occurring in the natural cycle of the parasite and those under the experimental conditions. Factors associated with the vector, such as mosquito salivary gland components, must be considered when interpreting the results (67). In addition, there is also an evidence arguing that *Leishmania* infection in mice by injecting millions of promastigotes subcutaneously in the hind footpad or tail ramp does not reproduce the natural form of the disease, where small number of metacyclic parasites are introduced during a sand fly bite (57).

### 3.1 Parasite factors

#### 3.1.1 Cutaneous leishmaniasis: experimental considerations

Different species of *Leishmania* including *L. major, L. tropica, L. mexicana, L. amazonensis, L. braziliensis* and *L. guyanensis* cause CL in the New and Old Worlds (**Table 1**). Several experimental studies have shown determining role of both parasite dose and site of inoculation in the outcome of cutaneous *Leishmania* infection in mice with different genetic backgrounds. In addition, type of immune response against the parasites leads to different pathology in the mice.

##### 3.1.1.1 Immune responses in control of the infection

Initial studies using the C57BL/6 and BALB/c mouse strains (**Table 2**) suggested that resistance (C57BL/6) or susceptibility (BALB/c) to *L. major* infection is associated with two types of Th1 and Th2 immune responses, respectively (8, 68, 69). It is noteworthy that the BALB/c susceptibility and C57BL/6 resistance model is primarily applicable to *L. major* infections, which have been the focus of most classical studies on the immune response in leishmaniasis. For other species of *Leishmania*, including the *Viannia* subgenus, the outcome of infection might be quite different; for example, typically most commonly used inbred mouse strains, including BALB/c mice, exhibit genetic resistance to *L. braziliensis* infection, resolving the infection within a few weeks. In contrast, the C57BL/6 strain develops a non-healing infection when infected with *L. mexicana* (**Table 2**) (70). For parasites in the V*iannia* subgenus, hamsters suggested as a more suitable model than mice, for the pathological study of localized and metastatic lesions (71).

**Table 2:** Comparison of immune responses in BALB/c and C57BL/6 to different *Leishmania* species.

*Leishmania* spp.	Human disease	Mouse disease				Reference
		C57BL/6 mice		BALB/c mice		
		Type of disease	Immune response	Type of disease	Immune response	
***L. major***	Self- healing CL	Self- healing lesion	Th1 (h.d.) 10^6 Transient Th2 (l.d.) 10^3	Visceralizing non-healing infection Resistance concomitant with parasite persistence Lesion development Lesion development	Th2 (h.d.) 10^6-10^7 Th1 (Very l.d.) 10^1-10^2 Th1/Th2 (l.d.)10^3 Th2>Th1 (Intermediate d.) 10^4-10^5	(119, 120, 139, 200–202) (56)
***L. amazonensis***	Self- healing CL or DCL	Non-healing infection	Th1 like but exacerbating disease	Non-healing infection	mixed Th1/Th2	(123–125)
***L. mexicana***	Self- healing American CL	Non-healing infection	TH1 and TH2	Non-healing infection	Th2	(94, 126)
***L. braziliensis***	Self- healing American CL and destructive MCL	non-ulcerated nodular lesion (10^6)	ND	non-ulcerated nodular lesion (10^6) Self- healing non-ulcerated nodular lesion (10^7) self-healing ulcerated lesion in ear dermis (10^5)	ND Th1 Th1	(127–129)
***L. infantum***	Typically cause VL,mostly children are affected, rarely cause CL	granulomatous response	mixed Th1/Th2 (h.d.) 10^7	Progressive VL, parasites clear from skin, gradual reduce from liver, persist in lymphnodes and spleen	infective dose is determinative; mixed Th1/Th2 (h.d.)(id) Th2 (h.d.)(sc) Th1 (l.d.)(sc OR id)	(130–132)
***L. donovani***	Cause VL in adults	Inhibition of granuloma formation	Lack of Th1 response or a Th2 response	Inhibition of granuloma formation	Lack of Th1 response or a Th2 response	(133–136)
***L. panamensis***	American CL	Self- healing lesion	ND	Non-healing infection Progressive disease with ulcerated lesion in ear dermis (10^5)	ND Mixed Th1/Th2	(137, 138)
L. tropica *	Typically antroponetic CL, rarely VL	Minimal pathology, persistent parasite over 1 year	Th1 response	Minimal pathology, persistent parasite over 1 year	Th1 response, IL-10 and TGFβ control the establishment of chronic infection	(203)

*C57BL/6 and BALB/c mice were infected in the ear dermis with 10^5 infectious stage, metacyclic promastigotes.

ND, not determined; h.d., high dose; l.d., low dose; id, intradermal; sc, subcutaneous. Bold letter highlights leishmania species.

Th1 response with production of IFNγ and IL-12 leads to lesion healing in resistant inbred mice (C57BL/6). Th1 type of cytokines particularly IFNγ, induce classically activated (M1) macrophages which initiate parasite killing. Macrophages produce two major anti-*Leishmania* components; reactive oxygen species (ROS) which is generated by respiratory burst during phagocytosis, and nitric oxide (NO), which is produced by Inducible nitric oxide synthase (iNOS) in response to IFNγ (4, 72, 73). Inhibition of the Th1 cells function by deleting the cytokines genes (IL-12, IFNγ, TNFα), their receptors (IFNγR), transcription factors (T-bet and STAT4) or co-stimulatory molecules (CD40–CD40L) lead to susceptibility to *L. major* infection (8). The role of ROS in controlling *Leishmania* infection in murine models varies depending on the parasite species and mouse strain. Unlike *L. major* infection, where ROS production plays a crucial role, in mouse models of *L. braziliensis* infection, ROS synthesis does not play a significant role in disease pathogenesis (74). Additionally, NO is a crucial factor in controlling *Leishmania* infection in mouse models and one of the key mechanisms through which IFN-γ enhances resistance to *L. major* infection is by stimulating iNOS expression in macrophages (75, 76) The role of iNOS/NO and ROS in human leishmaniasis remains less understood. While ROS production has been implicated in the killing of *L. braziliensis* by human macrophages, NO alone does not effectively control *L. braziliensis* infection in monocytes from CL patients *in vitro* (77). Studies have reported that NO production is undetectable in the supernatants of human macrophages infected with *L. infantum*; even though, *in vitro* inhibition of NO involved in parasite growth in these cells (78).

On the contrary, the Th2 response with the production of IL-4 leads to the expansion of the lesion and disseminated visceral infection in susceptible inbred mice (BALB/c). The activation of Th2 type cytokines, such as IL-4 and IL-13, drives the differentiation of alternatively activated macrophages (M2), which are characterized by elevated expression of *Arg1* and enhanced polyamine biosynthesis. This metabolic shift creates a favorable environment for amastigote proliferation within macrophages, ultimately contributing to disease progression (79, 80).

In BALB/c mice, lymphocytes of a third group, Th17, play a role in the extension of the lesions by producing cytokines such as IL-17 and IL-22 and infiltrating the polymorphonuclear cells into the infection site (81, 82). IL-17 is a potent pro-inflammatory cytokine that modulates immune responses by stimulating the production of various cytokines, including IL-6, IL-8, and GM-CSF, as well as chemokines such as CXCL1 and CXCL10. Additionally, IL-17 is essential for recruitment and activation of neutrophils at infection sites (83, 84), which are exploited by *Leishmania* parasites as temporary host cells to evade macrophage-mediated immune mechanisms (85). Th17 cells have also been shown to produce IL-21, IL-22 as well as IL-23, which is essential for the terminal differentiation of IL-17 producing effector T cells (86). In *L. major* infected BALB/c mice, both Th17 cells and neutrophils produce significantly higher amounts of IL-17 in comparison to cells from resistant C57BL/6 mice (81). *Leishmania*-infected DCs have been shown to induce IL-23 secretion, which in turn may help in the production of Th17 cells in BALB/c mice (81). In human leishmaniasis, an increased number of IL-17-expressing cells in lesions of *L. braziliensis*-infected patients has been associated with a higher cellular infiltrate (87). Additionally, elevated IL-17 levels have been observed in PBMC culture supernatants of active American CL cases compared to recovered patients (88).

Although Th1/Th2 paradigm is well established in the resistant strain C57BL/6 and the susceptible strain BALB/c, in human, this paradigm seems to be more complex and might be different in other mice strains (89–91). In murine models of *L. major* infection, a well-established paradigm suggests that successful healing involved activation of phagocytic cells, expansion of CD4+ Th1 cells, production of key cytokines such as IFN-γ, suppression of the Th2 response, and polarization of M1 macrophages. This ultimately enhances macrophage-mediated parasite-killing mechanisms, including NO synthesis (70). Over the past years, numerous studies have investigated the role of Th1/Th2 responses in human leishmaniasis, characterizing the phenotype of T cells and their polarized cytokines in lesions, cell cultures, or plasma of leishmaniasis patients (91–93). While cytokines like IFNγ are believed to play a role in controlling parasite infection during the healing process of human CL lesions (16), the classic Th1/Th2 polarization and IFN-γ/IL-4 interplays described in murine *L. major* infections do not fully translate to human disease or to infections caused by other species including the *Viannia* subgenus (70, 94).

In addition, existence of non-healing phenotypes in spite of a Th1 response (95, 96) along with evidence that some vaccines induce Th1 type cytokines without significantly effecting organ pathology (97, 98), or achieving protection without a strong Th1 response (99) suggests involvement of additional immune mechanisms. On the other hand, a delicate balance between pro- and anti-inflammatory cytokines is essential for an effective wound healing and the resolution of CL/MCL lesions. While a Th1 immune response is generally protective, an overproduction of pro-inflammatory cytokines can drive excessive immune cell recruitment to the infection site, exacerbating inflammation and ultimately leading to tissue destruction and damage. Elevated levels of IL-10 and transforming growth factor (TGF)-β help counteract this effect by suppressing pro-inflammatory cytokines, such as IFNγ, thereby mitigating inflammation and preventing tissue damage (100, 101). Studies have shown that IL-10 levels increase in PBMCs culture from patients with CL, playing a role in preventing immunopathology (102). MCL is typically characterized by an exaggerated inflammatory immune response, driven by an excessive reaction to the parasite, including elevated levels of specific antibodies and high concentrations of pro-inflammatory cytokines such as IFNγ, TNFα, and IL-6 (103). Parasites belonging to the *Leishmania* (*Viannia*) subgenus are the primary etiologic agents of human CL in the Americas. but among infected individuals, a small percentage progress to mucosal involvement. In MCL patients caused by *L. braziliensis*, increased production of IFNγ and TNFα coincides with reduced levels of IL-10 or IL-10 receptor expression in both PBMCs culture and lesion sites compared to patients without mucosal involvement (102, 104–106). CD4^+^ CD25^+^ regulatory T (T_REG_) cells contribute to Th1/Th2 immune modulation by producing cytokines such as TGFβ and IL-10, which suppress macrophage and dendritic cell activity, thereby limiting the release of inflammatory mediators at the *Leishmania* infection site (96, 107–110). An increased level of IL-17, produced by Th17 cells and polymorphonuclear (PMN) neutrophils, is another key characteristic of MCL lesions and PBMCs (88, 111). IL-17 is a potent pro-inflammatory cytokine contributing to excessive inflammation in the lesions (103).

Genetic studies in the cross between C57BL/6 and BALB/c mice revealed that QTLs (quantitative trait loci) *Lmr1* (*Leishmania major* resistance 1, -2, and -3 control *L. major* host response and wound healing independent of T helper cell responses and that a vigorous wound healing response was required for lesion resolution during *L. major* infection (112). Moreover, several studies using other mouse strains have shown that infection with *L. major* can induce several types of immune responses, which depends on the host genotype (45, 113). This is supported by the analysis of genetically engineered mice showing that some of cytokines (IFNγ, TNFα and IL-12) are necessary for defense against the parasite, whereas the others change their roles depending on genetic background, sub-strain of parasite and experimental design (9).

##### 3.1.1.2 Effect of parasite genotype, dose, and inoculation site

The outcome of the disease is influenced not only by different species of *Leishmania* (**Table 1**), but also by the genotypic variation among isolates of one species. Several studies showed that sub-strains of *L. major* species have different infectivity and virulence capability (64) in mouse model. Consequently, severities of symptoms, including the size and form of the lesions is different in every sub-strains of *Leishmania* (110, 114–117). Recent study using multilocus sequence typing (MLST) of seven housekeeping genes explored genetic variations of *Leishmania* strains isolated from atypical vs. typical CL patients in Iran. A high rate of genetic variations and heterozygosity was evident in *L. tropica* and *L. major* clinical strains (118). In addition, *Leishmania* clinical strains isolated from different CL patients demonstrated diverse immune responses and variable pathology in BALB/c mice (114, 116).

Scientists have long been puzzled over the ability of *L. major* Seidman strain (MHOM/SN/74/SD) to form non-healing cutaneous lesions in the face of a strong Th1 response in C57BL/6. It has been established that this phenomenon is due to ability of *L. major* sub-strain to infect a population of dermal macrophages in a mannose receptor 1, C-type 1 (MRC1/CD206)-dependent manner (54, 55). These macrophages exhibit an M2-polarized phenotype, making them permissive to infection and unable to effectively control the intracellular multiplication of *Leishmania* parasites.

In addition, a number of experimental studies have shown determining role of both parasite dose (119–122) and site of inoculation (57) in the outcome of cutaneous *Leishmania* infection in mice with different genetic backgrounds (57, 94, 123–140). In **Table 3** influence of inoculation site on the outcome of *L. major* infection in different mouse strains is outlined. As it is indicated, in BALB/c mice, subcutaneous (sc) injection of low doses (10^2 to 10^3) *L. major* did not induce lesion and was accompanied by stimulation of a Th1 type response. However, injecting doses higher than 10^5 caused a significant increase in the lesion size, associated with induction of a Th2 type of immune response (56). Although a low dose (10^2 parasite) initially caused pathology at the infection site in BALB/c mice, the lesion eventually healed (119). In the strain C57BL/6, a broad range of parasite doses from 10^2 to 10^7 elicited an effective Th1 type of immune response, which resulted in lesion healing, and only high doses (>10^6) were associated with lesion onset (120) (**Table 2**).

**Table 3:** Influence of the inoculation site on the output of *Leishmania major* infection and immune response of mice.

Inoculation Site	Mouse Strain	Lesion Size	Th1/Th2 Response	IFNγ/IL4 Ratio	Reference
**Hind footpad**	BALB/c	Highly S, nonhealing ulcer	Th2	Low	(57, 139, 140)
	C57BL/6	Resolving swelling by w4 pi	Th1	High	
	SWR	Small healing lesion	Th2	Low in w1, high in w8 pi	
**Ear pinna**	BALB/c	Highly S, nonhealing ulcer	Th2	Low in w5-10, baseline in w15 pi	
	C57BL/6	R, moderate swelling at w4, healed by w15	Th1	High in w5-10, baseline in w15 pi	
	C3H/HeN	Highly R	Th1	Low, small increase in w5 pi	
	CBA/H	Highly R	—	Low, same as control	
	DBA/2	Highly R	—	Low, same as control	
**Tail base**	BALB/c	Highly S, non-healing ulcer	Th2	Low in w5, baseline in w10 pi	
	C57BL/6	R, small swelling w4, healed by w10 pi	Th1/Th2	Low in w5-10, high in w15 pi	
	C3H/HeN	R, small lesion	Th1/Th2	Low in w5-10, very high in w15 pi	
	CBA/H	Highly R, small nodule w4 pi, healed w8 pi	—	Low	
	DBA/2	R, large ulcer w6 pi	—	Very Low	
	SWR	Highly S, non-healing ulcer	Th2	Low	

Mice infected with 1x10^4 in the ear pinna, or tail base (139) or with 5x10^5 in tail base, or hind footpad (57) or with 3x10^6 in hind footpad (140) of metacyclic promastigotes *of L. major* Friedlin strain.

W, week; pi, post infection; S, susceptible; R, resistant. Bold letters highlight the site of injection.

In resistant mice (C57BL/6) intradermal (id) injection of 100 metacyclic promastigotes in the ear can be used to partially mimic natural infection. In this case, two phases in infection course were defined: the first phase at early 4-6 weeks, in which the parasite replicates and the lesion is absent, and the second phase in which the lesion begins to develop while finally the lesion heals due to the predominance of the Th1 immune response (121).

The route of parasite inoculation has also been shown to have an influential role in the disease outcome (**Table 3**) (57). In the susceptible strain BALB/c, injection at any site induces non-healing lesions associated with an increase in Th2-type cytokine profiles (57). In resistant C57BL/6J mice, injection into the ear pinna induces a Th1 type of immune response with limited and self-healing lesion. However, injection into the tail ramp induces a Th2 type response, although the lesion eventually heals. Albeit, injection into the base of the tail in other resistant strains of mice such as DBA/2 and C3H/HeN caused partial or complete sensitivity to *Leishmania* infection (57). The clinical outcome of the infection and the severity of the lesions in these studies were not always associated with the type of immune response induced, as it is often seen in BALB/c or C57BL/6J mice (**Table 2**). Furthermore, the type of culture medium, number of passages, maintenance of the parasites, and percentage of metacyclic promastigotes influence the infectivity potential of the inoculum. That is why preparation of the inoculum need to be characterized and standardized well to reduce experimental variabilities. Together, the dose and the route of the parasite inoculation as well as parasite form determine the outcome of *Leishmania* infection in the experimental animal models, however, the genetic background of the host (mouse strain) and ultimately expanding of one of the two arms of either Th1 or Th2 immune responses is also of fundamental importance (reviewed in (122).

#### 3.1.2 Visceral leishmaniasis: experimental considerations

##### 3.1.2.1 Immune response in control of the infection

In contrast to a clear dichotomy of immune response against *L. major* infection in the strains BALB/c and C57BL/6, the Th1/Th2 concept does not explain susceptibility and resistance to visceral leishmaniasis caused by *L. infantum* (131) and *L. donovani* (133) in the mouse model.

Initial control and resolution of *L. donovani* hepatic infection in mice is accomplished within well-formed, mature tissue granulomas, which provide the microenvironment for intracellular *Leishmania* killing (136). The lack of a Th1 response or the presence of a Th2 response can inhibit granuloma formation in tissues of *L. donovani*-infected BALB/c (134, 135) and C57BL/6 mice (141). Experimental data indicated that *Il12* gene-deficient C57BL/6 mice are susceptible to *L. donovani*, but have diminished hepatic immunopathology associated with VL (141). The protective role of IL-12 in VL has been attributed to its ability to induce IFNγ production from NK and CD4+ T cells (142).

On the other hand, in the animal model of VL, the immune response is different depending on the infection in target organ (143). When *L. infantum* is injected intravenously (iv) into BALB/c mice, immune responses with different kinetics occur in the liver and spleen. In spleen, resident macrophages engage in leishmanicidal activity by increasing cytokines and producing nitric oxide (NO), and then parasite load is controlled (144). In the early infection, parasite replication is accompanied by inhibition of IFNγ and IL-2 secretion, and simultaneous increasing production of IL-10 and TGFβ by the spleen tissue inhibits macrophage activity causing further establishment of infection (144). In the later stages after 4 weeks of infection, the production of antigen-induced Th1 cytokines (IL-2 and IFNγ) stimulate leishmanicidal activity of macrophages leading to decrease in parasite burden. However, it seems that activity of CD4+CD25+ T_REG_ cells during the VL period, results in TGFβ production and establishment of a small number of persists in the spleen in *L. infantum*-infected BALB/c mice (145). In the liver of *L. donovani*-infected BALB/c and also C57BL/6 mice, the highest level of infection is shown in the 2^nd^ week that were largely eliminated by 4 weeks. It seems that development of immunity is due to formation of parasitized Kupffer cells granuloma leading to restriction of the infection and elimination of amastigotes from granulomas (146). Hence, an organ-specific immunity is suggested in VL: on one hand a protective immune response in the liver, which leads to the parasite elimination and on the other hand an ineffective immune response in the spleen that permits the parasite survival [Reviewed in (122)]. A detailed understanding of difference between these two types of immune responses in VL can be used to formulate new strategies in development of candidate vaccines or effective treatment against human VL.

##### 3.1.2.2 Effect of parasite genotype, dose and route of administration

Similar to *L.major*, there are intra-strain differences in the virulence of *L. infantum* isolated from different hosts belonging to the same zymodeme (MON-1) in the mouse model (147, 148). Strains with higher pathogenicity caused an increased parasite load in the spleen and liver of mice, which was associated with an enhanced TGFβ and a decreased IFNγ. Recently, biological differences or the behavior of Old and New World strains of *L. infantum (synonym L. chagasi)* has been investigated (148). The result showed differences in the infectivity potential of these two parasite strains in mice, the *L. infantum* Old World strain was more infective *in vivo* and *in vitro* than New World strain. The iNOS and arginine activities were also different in infected animals (148). Anyhow, the role of the host in VL virulence diversity is not fully understood and it is difficult to generalize the results from one strain (male BALB/c) to others (**Table 2**). Increasing numbers of experimental studies on animal model of VL suggest that the severity of the infection is associated with both dose of the parasite and the route of administration (130, 132, 149, 150). Usually, sc injection cause less infectivity than other routes of injection such as intradermal (id), intraperitoneal (ip) and intracardiac (ic). Typically, 10^5 parasite of *L. infantum* LIVT-1 strain with sc injection in mice was less infectious than iv injection, but this difference was not observed at higher doses (10^6) (122, 151–154) (**Table 2**).

Ic injection of *L. donovani* in BALB/c mice promotes development of a Th2 type of immune response associated with increased production of IL-4 and IgG1 as well as increased IL-10 level, which ultimately leads to progressive VL disease and parasite survival, especially in the spleen (149). Ic injection of *L. donovani* amastigotes causes progressive VL with immunosuppression characterized by defect in proliferative response of the splenic cells to *in vitro* stimulation with leishmanial antigen or the mitogen (155). Similarly, iv injection, especially in high doses, leads to the establishment of infection and parasite persistence in the liver and spleen (156). Protective immunity, characterized by granuloma formation in the liver and parasite clearance, was observed only in mice injected with a low dose of the parasite.

The usual routes of infection in the hamster model of VL are ic and ip. In experimental studies, *L. infantum* and *L. donovani*-infected Syrian hamsters (*Mesocricetus auratus*) often show typical clinical manifestations and pathological features of progressive VL, which are closely similar to active canine and human disease (152). However, the immunopathology of *L. donovani* infection in Syrian hamsters is extremely different from that observed in the murine models. Despite a robust Th1-like cytokine response, characterized by mRNA expression of IL-2, IFNγ, and TNFα, the hamster model exhibits increasing parasite replication in the liver, spleen, and bone marrow, indicating a possible dysfunction in macrophage effector activities. Notably, over the course of the infection, there is an absence of detectable inducible NO synthase in liver or spleen tissues, in contrast to what is usually observed in mice infections (153).

Following ic infection with *L. infantum*, hamsters display severe histopathological changes in both spleen and liver, where higher parasite burden are associated with different stages of granulomas formation with amastigotes in the liver, along with the disruption of the normal splenic architecture (154). Ic infection of BALB/c mice with *L. donovani* results in higher parasite loads in the liver and higher production of Th2 type cytokines IL-4 and IL-10 in the spleen in comparison to the sc, id or ip inoculation (149).

Overall, it seems that in the animal model of VL, using sc or id injection establishes an infection, which is more similar to natural disease. Moreover, a high dose can cause an effective infection in organs, and a low dose can induce a long-term immune response that might provide protection against *Leishmania* infection [Reviewed in (122)]. The mouse model of *L. infantum* infection replicates several features of human and canine VL, but Syrian hamsters exhibit severe clinical manifestations as usually seen in natural *Leishmania* infections (154). However, BALB/c mice remain the preferred model for studying VL pathogenesis and evaluating vaccine candidates, albeit they reflect self-healing or asymptomatic infections more accurately rather than progressive visceral disease. Unlike human VL, susceptible mouse strains fail to develop the full spectrum of progressive pathology. Moreover, disease severity in BALB/c mice varies based on inoculum size and infection route. In BALB/c mice, iv or id infection with *L. infantum* triggers organ-specific immune responses that shape disease progression. In the liver, an effective immunity with granuloma formation is formed to parasite elimination, whereas the spleen acts as a reservoir for persistent infection, highlighting its higher susceptibility to *L. infantum*. Understanding the mechanisms underlying this difference in organ specific immune responses may provide insights for developing targeted treatments for VL (154).

### 3.2 Host influencing factors

#### 3.2.1 Host sex

Host sex can differentially regulate susceptibility to leishmaniasis by modulating the immune response against the parasite [reviewed in (59)]. Usually, due to their easy handling female mice are used in research experiments, while in order to translate the data to human, both sexes must be equally considered.

Sexual dimorphisms have been observed in susceptibility to many infectious diseases including leishmaniasis. Sex may differentially affect pathology of various organs and its influence is modified by host’s hormonal status and genotype including sex chromosomes X and Y, as well as autosomal genes [reviewed in (59)]. Both DBA/2 female and male mice develop ulcerated lesions after infection with *L. major*, lesions heals in males, but not in females (157). On the contrary, DBA/2 female mice are highly resistant while males are susceptible to lesion development after infection with *L. mexicana* (157).

Influence of *Leishmania* species (*L. major* and *L. tropica*), sex and genetic background were analyzed in mouse strains BALB/c, STS, and recombinant congenic strains (RCS) CcS-3, CcS-5, CcS-11, CcS-12, CcS-16, CcS-18, and CcS-20. Each RCS contains a different random set of 12.5% genes from the parental “donor” strain STS and 87.5% genes from the “background” strain BALB/c (158). Infection by *L. major* induced larger skin lesions in males of strains CcS-3, CcS-5 and CcS-18, whereas no difference between males and females was observed in strains BALB/c, STS, CcS-11, CcS-12, CcS-16 and CcS-20. Females of strains BALB/c, CcS-11, CcS-16 and CcS-20 are more susceptible to development of skin lesions induced by *L. tropica*, whereas no sex bias was observed in strains STS, CcS-3, CcS-5, CcS-12 and CcS-18. Thus, sex differentially influences infection with *L. major* and *L. tropica*, however, observed differences are modified by the host genotype (42).

Interestingly, influence of sex on murine leishmaniasis in some genotypes is organ-specific. Strains BALB/c and CcS-11 did not exhibit any sex influence on lesion size induced by *L. major*, but males of strain CcS-11 contained more parasites in spleens than females, and males of both strains had much higher parasite load in lymph nodes (37).

#### 3.2.2 Host age

Age is an important factor that must be considered in studying infectious diseases using mice. Mice have a significantly shorter lifespan in comparison to humans; relatively, nine days in mice is almost equal to one year in human terms (159). Therefore, when designing an experiment, corresponding age of mice to human should be carefully estimated. With aging, frequencies of immune cells and expression of various immune receptors such as Toll-like receptors (TLRs) are changed, which can impair the host’s ability to combat infections, because they are part of pattern recognition receptors (PRRs) family that detect molecules from microbes and initiate immune responses (160). Clinical outcomes of diseases, which is dependent on host genetic, host immune response and environmental conditions, become more severe with aging. Several studies have proved that immune responses against *Leishmania* infection is altered with aging (61, 161). Aged C57BL/6 mice were more susceptible to *L. infantum* infection compared to young-infected mice, characterized by more parasite load in the spleen and liver (61). In contrast, in *L. major* infection experiments, macrophages derived from senescent C57BL/6 or BALB/c mice displayed similar anti-leishmanial activities compared to those from young mice. In addition, infection of resistant C57BL/6 mice with *L. major* revealed a similar course of footpad swelling between senescent and young mice. However, in susceptible BALB/c mice, senescent animals exhibited milder infections than their younger counterparts did, with 40–60% showing healing of lesion, reduced parasite dissemination, and a Th1 cell-mediated response, which was mainly due to spontaneous release of IL-12 by macrophages of aged mice. Interestingly, senescent BALB/c mice raised under specific-pathogen free (SPF) conditions showed neither resistance nor a Th1 response, indicating that exogenous microbial stimulation may also play a role in shaping immune responses during aging (63). Both conventionally kept BALB/c mice and SPF kept mice produced IL-12 cytokine but conventionally kept BALB/c mice were also infected with murine hepatitis virus (MHV). The spontaneous release of IL-12 due to aging and MHV infection induced Th1 response resulted protective response against *L. major* in the aged BALB/c mice (63). This result very well reflects the role of age and environmental conditions of animal models in experimental researches. In addition, this study highlights the potential impact of previous or co-infection in susceptibility of animal model, which usually is lacking in SPF condition.

#### 3.2.3 Host circadian rhythm

Pathological organism such as *Leishmania* can significantly alter circadian clock of the host, which can have a significant impact on the development of immune response against the infection. Change in circadian rhythm leads to an elevated inflammatory mediators that are not normally present in healthy individuals (162). In addition, infiltration and homing of circulating immune cells vary during the day/night; therefore, the timing of or the initiation of an experimental infection may lead to different outcomes depending on the time (163). For instance, the number of circulating monocytes and neutrophils are lower during the active (awake) phase while they go back to the peripheral organs such as bone marrow during the resting period (164). Therefore, altered circadian rhythm prior to an infection like *Leishmania*, can change the level of susceptibility to the disease and the immune responses (162). More importantly, mice and humans have a different circadian rhythm. In contrast to human, mice are nocturnal animals, being active during the night and resting during the day. Due to convenience, most experiments are started during the day, a time when the mice are supposed to be at rest. This situation can induce significant stress in mice that can influence the experiments outcome. Therefore, it might be of an importance to design experiments according to the biological clock of the animal models.

#### 3.2.4 Host microbiota

Increasing evidence shows that the gut microbiome hemostasis plays a crucial role in construction of an effective immune response against a disease. There are studies indicating that the host genetic regulates the host microbiome structure, however, inflammatory or infectious diseases along with environmental conditions can lead to an imbalance in the gut microbiome (165, 166). By analyzing the gut microbiome in mice with different genetic backgrounds, Mrázek et al. showed that the structure of gut microbiota significantly changes according to the genetic background of the host and *L. major* infection can change these components. Changes in gut microbiome can alter susceptibility to the disease (165, 167, 168). Different studies suggest re-construction of the host microbiota as a tool to create a basis for developing an effective therapeutic or vaccines against infectious diseases (165, 166).

#### 3.2.5 Host nutritional status

Host nutrition plays a major role in building effective immune responses against pathogens. Host diet has a direct effect on gut microbiome structure. In addition, unhealthy diet can enhance predisposition to cardiometabolic diseases such as obesity and diabetes as underlying conditions that make the host more susceptible to infections (169). Obesity influences clinical manifestations cutaneous leishmaniasis caused by *L. braziliensis* in humans and is associated with greater failure in therapy (170). Obese C57BL/6 mice are more susceptible to *L. major*, likely due to increased expansion of resident macrophages expressing CD206 (171). Insufficient nutrition intake or malnutrition characterized by deficit in protein, energy, zinc and iron disrupt anti-parasitic immunity during leishmaniasis (169, 172). It was shown that L. donovani disseminate faster from skin to visceral organs in malnourished mice (173). In addition it was reported that VL was significantly higher (more than three times) among malnourished people (174). Malnutrition lowers immunity against an infection by reducing immune cells and decreased inflammatory cytokines and enhanced anti-inflammatory cytokines production (172, 174). It is important to note that in experimental conditions, mice are often fed with chaw diet, which can be different from what they usually receive in the natural life. This artificial condition may have an impact on the *Leishmania* infection pathology.

#### 3.2.6 Host stress

Increasing evidence shows that mental health, stress and anxiety play an important role in modulating the immune responses against an infection. Leishmaniasis causes social exclusion/isolation, leading to an internalized self-stigma, stress, anxiety and depression (175, 176). A systemic review by Pires et al. showed that CL and PKDL patients and their family experienced high risk of mental illness, psychosocial morbidity and reduced quality of life (177). Moreover, ZCL were correlated with the loss of self-esteem and feelings of inferiority, which negatively correlates with age. Therefore, younger patients are psychologically more affected (178). Intestinally, low quality of life, anxiety and depression was more prevalent in female than male (177). *Leishmania* infection causes behavioral alterations and anxiety in mice (179). Stress and stigmatization in turn influence leishmaniasis outcomes (180, 181). Scientists are required to design and plan the experiments that have low level of stress. Construction noises, pollution, lack of experience with animal handling and intervention, not paying attention to circadian rhythm etc. can have significant impact on the experimental results.

#### 3.2.7 Vector influence

As it was mentioned earlier, the number of *Leishmania* parasites transmitted to the site of inoculation during natural transmission is very limited and is not comparable with the inoculum dose typically used in experimental infections. It was shown that salivary components and vector gut microbiota have a considerable role in infectivity and severity of leishmaniasis (67, 166, 182, 183). Salivary cDNA protein libraries has been constructed for 9 species of the genus *Phlebotomus* and 4 species of the genus *Lutzomyia* [reviewed in (184)]. More than 20 diverse proteins belonging to the different protein families have been identified in each cDNA library. Protein families that were detected in selected *Phlebotomus* as well as in *Lutzomyia* species are: antigen 5–related proteins, apyrases, odorant-binding proteins (D7-related proteins and PpSP15-like proteins), yellow-related proteins (YRPs), silk-related proteins, and lufaxin-like proteins (185). These proteins have anti-hemostatic, anti-inflammatory and immunomodulatory properties (184).

The most efficient way of experimental infection is inducing a natural infection by infected sand flies, however, technically, not every lab has a possibility to breed and maintain sand fly colonies, therefore, formulation of inoculum needs to be standardized and present the most similarity to the natural infection that usually occurs in human. Supplementing salivary gland lysate that contains saliva component and part of the vector microbiome might be a solution to increase the infectivity of *Leishmania* inoculum (186).

#### 3.2.8 Living conditions

Substantial evidence indicates that variations in laboratory mouse husbandry practices significantly contribute to the discrepancies observed in immune responses against pathogens, not only between mice and humans but also among experiments conducted at different institutes (19).

In the early history of laboratory mouse breeding, preventing contamination of mouse colonies by pathogens was a significant challenge until the filter-equipped cages were developed in the 1980s (187). The term “Specific Pathogen Free” (SPF), first introduced in the late 1950s, refers to mouse colonies that are devoid of specific pathogens, such as particular viruses, bacteria, and parasites (188). Although the list of these pathogens may vary, it typically includes those commonly shared with wild mice. The experimental exposure of SPF mice to specific microorganisms or modifications to their gut microbiome elicited distinct alterations in their immune responses to infection. Therefore, it is argued that manipulating the microbiome-host relationship in SPF models might greatly influence the application of findings to human health (189).

It was suggested that alterations in the living conditions of laboratory mice significantly influence the cellular profile of immune system (19). While the effector and tissue resident memory T cell repertoires were absent in laboratory mice, these cell populations were readily demonstrated in wild and pet store mice, naturally exposed to a broad spectrum of microbial environments. Interestingly, these immune cells were induced in laboratory mice after co-housing with pet store mice (190). It has been recommended that in the study of infectious diseases, colonies of inbred mice in controlled environments with microbiome resembling natural conditions are better suited for translating mouse immunology studies to human contexts (189).

## 4 Reproducibility of the data

A primary step toward improving the translation of mouse data to humans is to increase the reproducibility of mice experiments. The reproducibility crisis in experimental results remains a significant challenge in biomedical science. Considering aforementioned parameters not only improves the translational value of mouse experiments but also plays an important role in improving the reproducibility of animal experiments across different laboratories or even within the same laboratory. A critical step to increase the probability of data regeneration is the writing of a detailed standard operation procedure (SOP) for an experiment, ensuring it can be consistently followed by different investigators. In addition to the influencing parameters that were comprehensively discussed before, paying attention to the following routine practice should also be taken into consideration:

a. The animal experiments must be performed and repeated by trained staffs.
b. Factors such as the availability of materials and equipment, cleanliness of the facility and adherence to cleaning procedures, proper operation of the equipment, and handling of animals and the performance of interventions and routine checkups need to be standardized in the form of SOPs.
c. The breeding procedure or source of animal colony, along with factors such as age, sex, circadian clock and animal housing condition including bedding, temperature, humidity, food type, cage type (SPF or conventional), number of animals per cage, random distribution of the groups, sample size etc. must be strictly monitored and controlled.
d. Although ethical considerations may not directly influence experimental outcomes, adhering to ethical guidelines can help to unify most of the handling and housing procedures. Investigators must precisely and rigorously follow these ethical guidelines when designing, planning and conducting mouse experiments.

## 6 Conclusion remarks

Various animal models of CL and VL are employed in experimental studies to investigate the mechanisms of protective immunity and disease pathogenesis in *Leishmania* infections. The immune response to *Leishmania* is orchestrated through intricate regulatory networks, but the classic Th1/Th2 polarization observed in murine *L. major* infections do not fully translate to human disease or to infections caused by other species of *Leishmania* (70, 94). Therefore, there is no universal consensus on biomarkers of host susceptibility/resistance across human and experimental animal models, as immune responses can vary significantly depending on the host background and parasite factors as discussed above (reviewed in (4)).

Clinical manifestation of a disease such as leishmaniasis is a result of a complex cross talk between host genetic, immune responses and environmental conditions. Although, zoonotic types of leishmania infection naturally occurs in other mammalian hosts, development of mouse models has been an instrumental in furthering our understanding of the disease mechanism. Therefore, optimizing/adapting the influencing parameters in experiments to mimic infection in human is fundamental and will increase translational value of mouse data. Including more strains of mice with distinct genetic background as well as inbred strains to increase the genetic complexity may help to recapitulate the complexity of human genome in mice. In addition, cohosting the laboratory mice with pet mice instead of keeping them in extra clean conditions, optimizing and well characterization of parasite culture, and inclusion of sand fly saliva components in the parasite inoculum will help to induce an infection with closer clinical manifestation of human leishmaniasis. Furthermore, applying the current development in research such multiomics technologies and system biology along with characterizing environmental and host related parameters will help to recapitulate a disease condition closer to human leishmaniasis.

## References

[1] Mann S Frasca K Scherrer S Henao-Martinez AF Newman S Ramanan P. A review of leishmaniasis: current knowledge and future directions. Curr Trop Med Rep. (2021) 8:121–32. doi: 10.1007/s40475-021-00232-7 PMC7966913 33747716

[2] Burza S Croft SL Boelaert M. Leishmaniasis. Lancet. (2018) 392:951–70. doi: 10.1016/S0140-6736(18)31204-2 30126638

[3] Maroli M Feliciangeli MD Bichaud L Charrel RN Gradoni L. Phlebotomine sandflies and the spreading of leishmaniases and other diseases of public health concern. Med Vet Entomol. (2013) 27:123–47. doi: 10.1111/j.1365-2915.2012.01034.x 22924419

[4] Rostami MN Khamesipour A. Potential biomarkers of immune protection in human leishmaniasis. Med Microbiol Immunol. (2021) 210:81–100. doi: 10.1007/s00430-021-00703-8 33934238 PMC8088758

[5] Bogdan C. Mechanisms and consequences of persistence of intracellular pathogens: leishmaniasis as an example. Cell Microbiol. (2008) 10:1221–34. doi: 10.1111/j.1462-5822.2008.01146.x 18363880

[6] Terrazas CA Terrazas LI Gomez-Garcia L. Modulation of dendritic cell responses by parasites: a common strategy to survive. J BioMed Biotechnol. (2010) 2010:357106. doi: 10.1155/2010/357106 20204070 PMC2829630

[7] Loria-Cervera EN Andrade-Narvaez FJ. Animal models for the study of leishmaniasis immunology. Rev Inst Med Trop Sao Paulo. (2014) 56:1–11. doi: 10.1590/S0036-46652014000100001 24553602 PMC4085833

[8] Sacks D Noben-Trauth N. The immunology of susceptibility and resistance to Leishmania major in mice. Nat Rev Immunol. (2002) 2:845–58. doi: 10.1038/nri933 12415308

[9] Krayem I Lipoldova M. Role of host genetics and cytokines in Leishmania infection. Cytokine. (2021) 147:155244. doi: 10.1016/j.cyto.2020.155244 33059974

[10] Kobets T Grekov I Lipoldova M. Leishmaniasis: prevention, parasite detection and treatment. Curr Med Chem. (2012) 19:1443–74. doi: 10.2174/092986712799828300 22360481

[11] Boussoffara T Labidi I Trimeche M Chelbi I Dachraoui K Msallem N. LmCen(-/-) based vaccine is protective against canine visceral leishmaniasis following three natural exposures in Tunisia. NPJ Vaccines. (2025) 10:31. doi: 10.1038/s41541-025-01070-8 39952958 PMC11828870

[12] Singh OP Hasker E Sacks D Boelaert M Sundar S. Asymptomatic Leishmania infection: a new challenge for Leishmania control. Clin Infect Dis. (2014) 58:1424–9. doi: 10.1093/cid/ciu102 PMC4001287 24585564

[13] Pederiva MMC Santos SMD Rivarola LGS Guerreiro VJ Lopes KS Lima Junior M. Asymptomatic Leishmania infection in humans: A systematic review. J Infect Public Health. (2023) 16:286–94. doi: 10.1016/j.jiph.2022.12.021 36630836

[14] Nateghi-Rostami M Sohrabi Y. Memory T cells: promising biomarkers for evaluating protection and vaccine efficacy against leishmaniasis. Front Immunol. (2024) 15:1304696. doi: 10.3389/fimmu.2024.1304696 38469319 PMC10925770

[15] Nateghi Rostami M Keshavarz H Khamesipour A. Immune response of BALB/c mice against an experimental vaccine of Alum precipitated autoclaved Leishmania major (Alum-ALM) mixed with BCG or Mycobacterium vaccae. Trop Biomed. (2010) 27:89–102. 20562818

[16] Miramin-Mohammadi A Javadi A Eskandari SE Mortazavi H Rostami MN Khamesipour A. Immune response in cutaneous leishmaniasis patients with healing vs. non-healing lesions. Iran J Microbiol. (2020) 12:249–55. doi: 10.18502/ijm.v12i3.3243 PMC7340611 32685122

[17] Lera-Nonose D De Oliveira LF Brustolin A Santos TS Oyama J Ramos-Milare A. Genetic variations in the human immune system influence susceptibility to tegumentary leishmaniasis: a systematic review and meta-analysis. Expert Rev Clin Immunol. (2021) 17:513–37. doi: 10.1080/1744666X.2021.1906650 33749481

[18] Bharati K. Human genetic polymorphism and Leishmaniasis. Infection Genet evolution: J Mol Epidemiol evolutionary Genet Infect diseases. (2022) 98:105203. doi: 10.1016/j.meegid.2021.105203 34990851

[19] Masopust D Sivula CP Jameson SC. Of mice, dirty mice, and men: using mice to understand human immunology. J Immunol. (2017) 199:383–8. doi: 10.4049/jimmunol.1700453 PMC5512602 28696328

[20] Mikhail JW Mansour NS. Leishmania donovani: therapeutic and prophylaciic action of antimony dextran glycoside (RL-712) in the golden hamster. Exp Parasitol. (1975) 37:348–52. doi: 10.1016/0014-4894(75)90002-8 1126418

[21] Keenan CM Hendricks LD Lightner L Webster HK Johnson AJ. Visceral leishmaniasis in the German shepherd dog. I. Infection, clinical disease, and clinical pathology. Vet Pathol. (1984) 21:74–9. doi: 10.1177/030098588402100113 6710816

[22] Chapman WL Jr. Hanson WL Hendricks LD. Toxicity and efficacy of the antileishmanial drug meglumine antimoniate in the owl monkey (Aotus trivirgatus). J Parasitol. (1983) 69:1176–7. doi: 10.2307/3280894 6674468

[23] Hommel M Jaffe CL Travi B Milon G. Experimental models for leishmaniasis and for testing anti-leishmanial vaccines. Ann Trop Med Parasitol. (1995) 89 Suppl 1:55–73. doi: 10.1080/00034983.1995.11813015 8745928

[24] Courret N Lang T Milon G Antoine JC. Intradermal inoculations of low doses of Leishmania major and Leishmania amazonensis metacyclic promastigotes induce different immunoparasitic processes and status of protection in BALB/c mice. Int J Parasitol. (2003) 33:1373–83. doi: 10.1016/S0020-7519(03)00179-6 14527520

[25] Behforouz NC Wenger CD Mathison BA. Prophylactic treatment of BALB/c mice with cyclosporine A and its analog B-5-49 enhances resistance to Leishmania major. J Immunol. (1986) 136:3067–75. doi: 10.4049/jimmunol.136.8.3067 3958489

[26] Van den Kerkhof M Mabille D Chatelain E Mowbray CE Braillard S Hendrickx S. *In vitro* and *in vivo* pharmacodynamics of three novel antileishmanial lead series. Int J Parasitol Drugs Drug Resist. (2018) 8:81–6. doi: 10.1016/j.ijpddr.2018.01.006 PMC6114106 29425734

[27] van der Ende J Schallig H. Leishmania animal models used in drug discovery: A systematic review. Anim (Basel). (2023) 13(10):1650. doi: 10.3390/ani13101650 PMC10215483 37238080

[28] Tegazzini D Diaz R Aguilar F Pena I Presa JL Yardley V. A Replicative *In Vitro* Assay for Drug Discovery against Leishmania donovani. Antimicrob Agents Chemother. (2016) 60:3524–32. doi: 10.1128/AAC.01781-15 PMC4879429 27021313

[29] Fortin A Caridha DP Leed S Ngundam F Sena J Bosschaerts T. Direct comparison of the efficacy and safety of oral treatments with oleylphosphocholine (OlPC) and miltefosine in a mouse model of L. major cutaneous leishmaniasis. PloS Negl Trop Dis. (2014) 8:e3144. doi: 10.1371/journal.pntd.0003144 25210745 PMC4161350

[30] Garcia Bustos MF Barrio A Prieto GG de Raspi EM Cimino RO Cardozo RM. *In vivo* antileishmanial efficacy of miltefosine against Leishmania (Leishmania) amazonensis. J Parasitol. (2014) 100:840–7. doi: 10.1645/13-376.1 25014108

[31] Jaafari MR Bavarsad N Bazzaz BS Samiei A Soroush D Ghorbani S. Effect of topical liposomes containing paromomycin sulfate in the course of Leishmania major infection in susceptible BALB/c mice. Antimicrob Agents Chemother. (2009) 53:2259–65. doi: 10.1128/AAC.01319-08 PMC2687252 19223613

[32] Neira LF Mantilla JC Escobar P. Anti-leishmanial activity of a topical miltefosine gel in experimental models of New World cutaneous leishmaniasis. J Antimicrob Chemother. (2019) 74:1634–41. doi: 10.1093/jac/dkz049 30815688

[33] Herrera L Llanes A Alvarez J Degracia K Restrepo CM Rivera R. Antileishmanial activity of a new chloroquine analog in an animal model of Leishmania panamensis infection. Int J Parasitol Drugs Drug Resist. (2020) 14:56–61. doi: 10.1016/j.ijpddr.2020.08.002 32950020 PMC7502791

[34] Mears ER Modabber F Don R Johnson GE. A review: the current *in vivo* models for the discovery and utility of new anti-leishmanial drugs targeting cutaneous leishmaniasis. PloS Negl Trop Dis. (2015) 9:e0003889. doi: 10.1371/journal.pntd.0003889 26334763 PMC4559374

[35] Sakthianandeswaren A Foote SJ Handman E. The role of host genetics in leishmaniasis. Trends Parasitol. (2009) 25:383–91. doi: 10.1016/j.pt.2009.05.004 19617002

[36] Vladimirov V Badalova J Svobodova M Havelkova H Hart AA Blazkova H. Different genetic control of cutaneous and visceral disease after Leishmania major infection in mice. Infect Immun. (2003) 71:2041–6. doi: 10.1128/IAI.71.4.2041-2046.2003 PMC152088 12654824

[37] Kurey I Kobets T Havelkova H Slapnickova M Quan L Trtkova K. Distinct genetic control of parasite elimination, dissemination, and disease after Leishmania major infection. Immunogenetics. (2009) 61:619–33. doi: 10.1007/s00251-009-0392-9 PMC2744819 19705113

[38] Krayem I Sohrabi Y Havelkova H Gusareva ES Strnad H Cepickova M. Functionally distinct regions of the locus Leishmania major response 15 control IgE or IFNgamma level in addition to skin lesions. Front Immunol. (2023) 14:1145269. doi: 10.3389/fimmu.2023.1145269 37600780 PMC10437074

[39] Krayem I Sohrabi Y Javorkova E Volkova V Strnad H Havelkova H. Genetic influence on frequencies of myeloid-derived cell subpopulations in mouse. Front Immunol. (2021) 12:760881. doi: 10.3389/fimmu.2021.760881 35154069 PMC8826059

[40] Sohrabi Y Volkova V Kobets T Havelkova H Krayem I Slapnickova M. Genetic regulation of guanylate-binding proteins 2b and 5 during leishmaniasis in mice. Front Immunol. (2018) 9:130. doi: 10.3389/fimmu.2018.00130 29467757 PMC5808352

[41] Lipoldova M Demant P. Genetic susceptibility to infectious disease: lessons from mouse models of leishmaniasis. Nat Rev Genet. (2006) 7:294–305. doi: 10.1038/nrg1832 16543933

[42] Kobets T Havelkova H Grekov I Volkova V Vojtiskova J Slapnickova M. Genetics of host response to Leishmania tropica in mice - different control of skin pathology, chemokine reaction, and invasion into spleen and liver. PloS Negl Trop Dis. (2012) 6:e1667. doi: 10.1371/journal.pntd.0001667 22679519 PMC3367980

[43] Havelkova H Badalova J Svobodova M Vojtikova J Kurey I Vladimirov V. Genetics of susceptibility to leishmaniasis in mice: four novel loci and functional heterogeneity of gene effects. Genes Immun. (2006) 7:220–33. doi: 10.1038/sj.gene.6364290 16511555

[44] Sohrabi Y Havelkova H Kobets T Sima M Volkova V Grekov I. Mapping the genes for susceptibility and response to Leishmania tropica in mouse. PloS Negl Trop Dis. (2013) 7:e2282. doi: 10.1371/journal.pntd.0002282 23875032 PMC3708836

[45] Lipoldova M Svobodova M Havelkova H Krulova M Badalova J Nohynkova E. Mouse genetic model for clinical and immunological heterogeneity of leishmaniasis. Immunogenetics. (2002) 54:174–83. doi: 10.1007/s00251-002-0439-7 12073146

[46] Kobets T Cepickova M Volkova V Sohrabi Y Havelkova H Svobodova M. Novel loci controlling parasite load in organs of mice infected with leishmania major, their interactions and sex influence. Front Immunol. (2019) 10:1083. doi: 10.3389/fimmu.2019.01083 31231359 PMC6566641

[47] Badalova J Svobodova M Havelkova H Vladimirov V Vojtiskova J Engova J. Separation and mapping of multiple genes that control IgE level in Leishmania major infected mice. Genes Immun. (2002) 3:187–95. doi: 10.1038/sj.gene.6363838 12058253

[48] Lipoldova M Svobodova M Krulova M Havelkova H Badalova J Nohynkova E. Susceptibility to Leishmania major infection in mice: multiple loci and heterogeneity of immunopathological phenotypes. Genes Immun. (2000) 1:200–6. doi: 10.1038/sj.gene.6363660 11196712

[49] Vidal SM Malo D Vogan K Skamene E Gros P. Natural resistance to infection with intracellular parasites: isolation of a candidate for Bcg. Cell. (1993) 73:469–85. doi: 10.1016/0092-8674(93)90135-d 8490962

[50] Bucheton B Abel L Kheir MM Mirgani A El-Safi SH Chevillard C. Genetic control of visceral leishmaniasis in a Sudanese population: candidate gene testing indicates a linkage to the NRAMP1 region. Genes Immun. (2003) 4:104–9. doi: 10.1038/sj.gene.6363927 12618857

[51] Sakthianandeswaren A Curtis JM Elso C Kumar B Baldwin TM Lopaticki S. Fine mapping of Leishmania major susceptibility Locus lmr2 and evidence of a role for Fli1 in disease and wound healing. Infect Immun. (2010) 78:2734–44. doi: 10.1128/IAI.00126-10 PMC2876540 20368343

[52] Castellucci L Jamieson SE Miller EN de Almeida LF Oliveira J Magalhaes A. FLI1 polymorphism affects susceptibility to cutaneous leishmaniasis in Brazil. Genes Immun. (2011) 12:589–94. doi: 10.1038/gene.2011.37 PMC3297968 21633373

[53] Tao L Reese TA. Making mouse models that reflect human immune responses. Trends Immunol. (2017) 38:181–93. doi: 10.1016/j.it.2016.12.007 28161189

[54] Lee SH Charmoy M Romano A Paun A Chaves MM Cope FO. Mannose receptor high, M2 dermal macrophages mediate nonhealing Leishmania major infection in a Th1 immune environment. J Exp Med. (2018) 215:357–75. doi: 10.1084/jem.20171389 PMC5748861 29247046

[55] Sohrabi Y Lipoldova M. Mannose receptor and the mystery of nonhealing leishmania major infection. Trends Parasitol. (2018) 34:354–6. doi: 10.1016/j.pt.2018.03.006 29650366

[56] Menon JN Bretscher PA. Parasite dose determines the Th1/Th2 nature of the response to Leishmania major independently of infection route and strain of host or parasite. Eur J Immunol. (1998) 28:4020–8. doi: 10.1002/(SICI)1521-4141(199812)28:12<4020::AID-IMMU4020>3.0.CO;2-3 9862338

[57] Baldwin TM Elso C Curtis J Buckingham L Handman E. The site of Leishmania major infection determines disease severity and immune responses. Infect Immun. (2003) 71:6830–4. doi: 10.1128/IAI.71.12.6830-6834.2003 PMC308923 14638769

[58] Lockard RD Wilson ME Rodriguez NE. Sex-related differences in immune response and symptomatic manifestations to infection with leishmania species. J Immunol Res. (2019) 2019:4103819. doi: 10.1155/2019/4103819 30756088 PMC6348913

[59] Lipoldova M Demant P. Gene-specific sex effects on susceptibility to infectious diseases. Front Immunol. (2021) 12:712688. doi: 10.3389/fimmu.2021.712688 34721380 PMC8553003

[60] Vom Steeg LG Klein SL. Sex and sex steroids impact influenza pathogenesis across the life course. Semin Immunopathol. (2019) 41:189–94. doi: 10.1007/s00281-018-0718-5 PMC6370518 30298431

[61] Loureiro Salgado C Mendez Corea AF Covre LP De Matos Guedes HL Falqueto A Gomes DCO. Ageing impairs protective immunity and promotes susceptibility to murine visceral leishmaniasis. Parasitology. (2022) 149:1249–56. doi: 10.1017/S0031182022000828 PMC11010576 35670372

[62] Oliveira MR Tafuri WL Afonso LC Oliveira MA Nicoli JR Vieira EC. Germ-free mice produce high levels of interferon-gamma in response to infection with Leishmania major but fail to heal lesions. Parasitology. (2005) 131:477–88. doi: 10.1017/S0031182005008073 16174412

[63] Ehrchen J Sindrilaru A Grabbe S Schonlau F Schlesiger C Sorg C. Senescent BALB/c mice are able to develop resistance to Leishmania major infection. Infect Immun. (2004) 72:5106–14. doi: 10.1128/IAI.72.9.5106-5114.2004 PMC517419 15322004

[64] Sadlova J Svobodova M Volf P. Leishmania major: effect of repeated passages through sandfly vectors or murine hosts. Ann Trop Med Parasitol. (1999) 93:599–611. doi: 10.1080/00034989958104 10707105

[65] Grekov I Svobodova M Nohynkova E Lipoldova M. Preparation of highly infective Leishmania promastigotes by cultivation on SNB-9 biphasic medium. J Microbiol Methods. (2011) 87:273–7. doi: 10.1016/j.mimet.2011.08.012 21889549

[66] Nemati Haravani T Parvizi P Hejazi SH Sedaghat MM Eskandarian A Nateghi Rostami M. Evaluation of expression variations in virulence-related genes of Leishmania major after several culture passages compared with Phlebotomus papatasi isolated promastigotes. PloS One. (2023) 18:e0284240. doi: 10.1371/journal.pone.0284240 37053214 PMC10101501

[67] Serafim TD Coutinho-Abreu IV Dey R Kissinger R Valenzuela JG Oliveira F. Leishmaniasis: the act of transmission. Trends Parasitol. (2021) 37:976–87. doi: 10.1016/j.pt.2021.07.003 34389215

[68] Heinzel FP Sadick MD Holaday BJ Coffman RL Locksley RM. Reciprocal expression of interferon gamma or interleukin 4 during the resolution or progression of murine leishmaniasis. Evidence for expansion of distinct helper T cell subsets. J Exp Med. (1989) 169:59–72. doi: 10.1084/jem.169.1.59 2521244 PMC2189187

[69] Sadick MD Locksley RM Tubbs C Raff HV. Murine cutaneous leishmaniasis: resistance correlates with the capacity to generate interferon-gamma in response to Leishmania antigens *in vitro* . J Immunol. (1986) 136:655–61. doi: 10.4049/jimmunol.136.2.655 3079789

[70] Soong L Henard CA Melby PC. Immunopathogenesis of non-healing American cutaneous leishmaniasis and progressive visceral leishmaniasis. Semin Immunopathol. (2012) 34:735–51. doi: 10.1007/s00281-012-0350-8 PMC4111229 23053396

[71] Martinez JE Valderrama L Gama V Leiby DA Saravia NG. Clonal diversity in the expression and stability of the metastatic capability of Leishmania guyanensis in the golden hamster. J Parasitol. (2000) 86:792–9. doi: 10.1645/0022-3395(2000)086[0792:CDITEA]2.0.CO;2 10958458

[72] Bogdan C Rollinghoff M Diefenbach A. Reactive oxygen and reactive nitrogen intermediates in innate and specific immunity. Curr Opin Immunol. (2000) 12:64–76. doi: 10.1016/S0952-7915(99)00052-7 10679404

[73] Van Assche T Deschacht M da Luz RA Maes L Cos P. Leishmania-macrophage interactions: insights into the redox biology. Free Radic Biol Med. (2011) 51:337–51. doi: 10.1016/j.freeradbiomed.2011.05.011 21620959

[74] Rocha FJ Schleicher U Mattner J Alber G Bogdan C. Cytokines, signaling pathways, and effector molecules required for the control of Leishmania (Viannia) Braziliensis in mice. Infect Immun. (2007) 75:3823–32. doi: 10.1128/IAI.01335-06 PMC1951993 17517868

[75] Mattner J Schindler H Diefenbach A Rollinghoff M Gresser I Bogdan C. Regulation of type 2 nitric oxide synthase by type 1 interferons in macrophages infected with Leishmania major. Eur J Immunol. (2000) 30:2257–67. doi: 10.1002/1521-4141(2000)30:8<2257::AID-IMMU2257>3.0.CO;2-U 10940917

[76] Wei XQ Charles IG Smith A Ure J Feng GJ Huang FP. Altered immune responses in mice lacking inducible nitric oxide synthase. Nature. (1995) 375:408–11. doi: 10.1038/375408a0 7539113

[77] Carneiro PP Conceicao J Macedo M Magalhaes V Carvalho EM Bacellar O. The role of nitric oxide and reactive oxygen species in the killing of leishmania Braziliensis by monocytes from patients with cutaneous leishmaniasis. PloS One. (2016) 11:e0148084. doi: 10.1371/journal.pone.0148084 26840253 PMC4739692

[78] Gantt KR Goldman TL McCormick ML Miller MA Jeronimo SM Nascimento ET. Oxidative responses of human and murine macrophages during phagocytosis of Leishmania chagasi. J Immunol. (2001) 167:893–901. doi: 10.4049/jimmunol.167.2.893 11441096

[79] Tomiotto-Pellissier F Bortoleti B Assolini JP Goncalves MD Carloto ACM Miranda-Sapla MM. Macrophage polarization in leishmaniasis: broadening horizons. Front Immunol. (2018) 9:2529. doi: 10.3389/fimmu.2018.02529 30429856 PMC6220043

[80] Gaur U Roberts SC Dalvi RP Corraliza I Ullman B Wilson ME. An effect of parasite-encoded arginase on the outcome of murine cutaneous leishmaniasis. J Immunol. (2007) 179:8446–53. doi: 10.4049/jimmunol.179.12.8446 18056391

[81] Lopez Kostka S Dinges S Griewank K Iwakura Y Udey MC von Stebut E. IL-17 promotes progression of cutaneous leishmaniasis in susceptible mice. J Immunol. (2009) 182:3039–46. doi: 10.4049/jimmunol.0713598 PMC2658650 19234200

[82] Nateghi Rostami M Seyyedan Jasbi E Khamesipour A Mohammadi AM. Tumour Necrosis Factor-alpha (TNF-alpha) and its soluble receptor type 1 (sTNFR I) in human active and healed leishmaniases. Parasite Immunol. (2016) 38:255–60. doi: 10.1111/pim.2016.38.issue-4 26813918

[83] Bettelli E Korn T Kuchroo VK. Th17: the third member of the effector T cell trilogy. Curr Opin Immunol. (2007) 19:652–7. doi: 10.1016/j.coi.2007.07.020 PMC2288775 17766098

[84] Banerjee A Bhattacharya P Joshi AB Ismail N Dey R Nakhasi HL. Role of pro-inflammatory cytokine IL-17 in Leishmania pathogenesis and in protective immunity by Leishmania vaccines. Cell Immunol. (2016) 309:37–41. doi: 10.1016/j.cellimm.2016.07.004 27444130

[85] van Zandbergen G Klinger M Mueller A Dannenberg S Gebert A Solbach W. Cutting edge: neutrophil granulocyte serves as a vector for Leishmania entry into macrophages. J Immunol. (2004) 173:6521–5. doi: 10.4049/jimmunol.173.11.6521 15557140

[86] McGeachy MJ Chen Y Tato CM Laurence A Joyce-Shaikh B Blumenschein WM. The interleukin 23 receptor is essential for the terminal differentiation of interleukin 17-producing effector T helper cells *in vivo* . Nat Immunol. (2009) 10:314–24. doi: 10.1038/ni.1698 PMC2945605 19182808

[87] Bacellar O Faria D Nascimento M Cardoso TM Gollob KJ Dutra WO. Interleukin 17 production among patients with American cutaneous leishmaniasis. J Infect diseases. (2009) 200:75–8. doi: 10.1086/599380 PMC2732405 19476435

[88] Souza MA Castro MC Oliveira AP Almeida AF Reis LC Silva CJ. American tegumentary leishmaniasis: cytokines and nitric oxide in active disease and after clinical cure, with or without chemotherapy. Scand J Immunol. (2012) 76:175–80. doi: 10.1111/j.1365-3083.2012.02717.x 22537157

[89] Darzi F Davoudian R Nateghi Rostami M. Differential inflammatory responses associated with Leishmania major and L tropica in culture. Parasite Immunol. (2021) 43:e12841. doi: 10.1111/pim.12841 33914948

[90] Keshavarz Valian H Nateghi Rostami M Tasbihi M Miramin Mohammadi A Eskandari SE Sarrafnejad A. CCR7+ central and CCR7- effector memory CD4+ T cells in human cutaneous leishmaniasis. J Clin Immunol. (2013) 33:220–34. doi: 10.1007/s10875-012-9788-7 22990666

[91] Khamesipour A Nateghi Rostami M Tasbihi M Miramin Mohammadi A Shahrestani T Sarrafnejad A. Phenotyping of circulating CD8(+) T cell subsets in human cutaneous leishmaniasis. Microbes infection/Institut Pasteur. (2012) 14:702–11. doi: 10.1016/j.micinf.2012.02.006 22421108

[92] Nateghi Rostami M Seyyedan Jasbi E Khamesipour A Miramin Mohammadi A. Plasma levels of tumor necrosis factor-alpha (TNF-alpha), TNF-alpha soluble receptor type 1 (sTNFR I) and IL-22 in human leishmaniasis. Trop Biomed. (2015) 32:478–84. 26695208

[93] Miramin-Mohammadi A Javadi A Eskandari SE Nateghi-Rostami M Khamesipour A. Immune Responses in Cutaneous Leishmaniasis: *In vitro* Thelper1/Thelper2 Cytokine Profiles Using Live Versus Killed Leishmania major. J Arthropod Borne Dis. (2021) 15:126–35. doi: 10.18502/jad.v15i1.6491 PMC8271235 34277861

[94] McMahon-Pratt D Alexander J. Does the Leishmania major paradigm of pathogenesis and protection hold for New World cutaneous leishmaniases or the visceral disease? Immunol Rev. (2004) 201:206–24. doi: 10.1111/j.0105-2896.2004.00190.x 15361243

[95] Sacks D Anderson C. Re-examination of the immunosuppressive mechanisms mediating non-cure of Leishmania infection in mice. Immunol Rev. (2004) 201:225–38. doi: 10.1111/j.0105-2896.2004.00185.x 15361244

[96] Fromm PD Kling JC Remke A Bogdan C Korner H. Fatal leishmaniasis in the absence of TNF despite a strong th1 response. Front Microbiol. (2015) 6:1520. doi: 10.3389/fmicb.2015.01520 26834705 PMC4722107

[97] Campos-Neto A. Anti-leishmania vaccine. In: Farrel JP, editor. Leishmania. Kluwer Academic Publishers, Boston (2002). p. 169–90.

[98] Sjölander A Baldwin TM Curtis JM Bengtsson KL Handman E. Vaccination with recombinant Parasite Surface Antigen 2 from Leishmania major induces a Th1 type of immune response but does not protect against infection. Vaccine. (1998) 16:2077–84. doi: 10.1016/S0264-410X(98)00075-9 9796067

[99] Uzonna JE Spath GF Beverley SM Scott P. Vaccination with phosphoglycan-deficient Leishmania major protects highly susceptible mice from virulent challenge without inducing a strong Th1 response. J Immunol. (2004) 172:3793–7. doi: 10.4049/jimmunol.172.6.3793 15004184

[100] Rocha PN Almeida RP Bacellar O de Jesus AR Filho DC Filho AC. Down-regulation of Th1 type of response in early human American cutaneous leishmaniasis. J Infect diseases. (1999) 180:1731–4. doi: 10.1086/jid.1999.180.issue-5 10515843

[101] Oliveira WN Ribeiro LE Schrieffer A MaChado P Carvalho EM Bacellar O. The role of inflammatory and anti-inflammatory cytokines in the pathogenesis of human tegumentary leishmaniasis. Cytokine. (2014) 66:127–32. doi: 10.1016/j.cyto.2013.12.016 PMC4047562 24485388

[102] Gaze ST Dutra WO Lessa M Lessa H Guimaraes LH Jesus AR. Mucosal leishmaniasis patients display an activated inflammatory T-cell phenotype associated with a nonbalanced monocyte population. Scand J Immunol. (2006) 63:70–8. doi: 10.1111/j.1365-3083.2005.01707.x 16398703

[103] Scott P Novais FO. Cutaneous leishmaniasis: immune responses in protection and pathogenesis. Nat Rev Immunol. (2016) 16:581–92. doi: 10.1038/nri.2016.72 27424773

[104] Faria DR Gollob KJ Barbosa J Jr. Schriefer A MaChado PR Lessa H. Decreased in *situ* expression of interleukin-10 receptor is correlated with the exacerbated inflammatory and cytotoxic responses observed in mucosal leishmaniasis. Infect Immun. (2005) 73:7853–9. doi: 10.1128/IAI.73.12.7853-7859.2005 PMC1307048 16299275

[105] Bacellar O Lessa H Schriefer A MaChado P Ribeiro de Jesus A Dutra WO. Up-regulation of Th1-type responses in mucosal leishmaniasis patients. Infect Immun. (2002) 70:6734–40. doi: 10.1128/IAI.70.12.6734-6740.2002 PMC132996 12438348

[106] Nylen S Eidsmo L. Tissue damage and immunity in cutaneous leishmaniasis. Parasite Immunol. (2012) 34:551–61. doi: 10.1111/pim.2012.34.issue-12 23009296

[107] Dubie T Mohammed Y. Review on the role of host immune response in protection and immunopathogenesis during cutaneous leishmaniasis infection. J Immunol Res. (2020) 2020:2496713. doi: 10.1155/2020/2496713 32656269 PMC7320295

[108] Belkaid Y Piccirillo CA Mendez S Shevach EM Sacks DL. CD4+CD25+ regulatory T cells control Leishmania major persistence and immunity. Nature. (2002) 420:502–7. doi: 10.1038/nature01152 12466842

[109] Belkaid Y. The role of CD4(+)CD25(+) regulatory T cells in Leishmania infection. Expert Opin Biol Ther. (2003) 3:875–85. doi: 10.1517/14712598.3.6.875 12943446

[110] Attia H Sghaier MR Bali A Guerfali FZ Chlif S Atri C. Intra-specific diversity of leishmania major isolates: A key determinant of Tunisian zoonotic cutaneous leishmaniasis clinical polymorphism. Microorganisms. (2022) 10(3):505. doi: 10.3390/microorganisms10030505 35336081 PMC8955835

[111] Boaventura VS Santos CS Cardoso CR de Andrade J Dos Santos WL Clarencio J. Human mucosal leishmaniasis: neutrophils infiltrate areas of tissue damage that express high levels of Th17-related cytokines. Eur J Immunol. (2010) 40:2830–6. doi: 10.1002/eji.200940115 20812234

[112] Elso CM Roberts LJ Smyth GK Thomson RJ Baldwin TM Foote SJ. Leishmaniasis host response loci (lmr1-3) modify disease severity through a Th1/Th2-independent pathway. Genes Immun. (2004) 5:93–100. doi: 10.1038/sj.gene.6364042 14668789

[113] Babay BE Louzir H Kebaier C Boubaker S Dellagi K Cazenave PA. Inbred strains derived from feral mice reveal new pathogenic mechanisms of experimental leishmaniasis due to Leishmania major. Infect Immun. (2004) 72:4603–11. doi: 10.1128/IAI.72.8.4603-4611.2004 PMC470675 15271920

[114] Kebaier C Louzir H Chenik M Ben Salah A Dellagi K. Heterogeneity of wild Leishmania major isolates in experimental murine pathogenicity and specific immune response. Infect Immun. (2001) 69:4906–15. doi: 10.1128/IAI.69.8.4906-4915.2001 PMC98581 11447167

[115] Alimohammadian MH Darabi H Ajdary S Khaze V Torkabadi E. Genotypically distinct strains of Leishmania major display diverse clinical and immunological patterns in BALB/c mice. Infection Genet evolution: J Mol Epidemiol evolutionary Genet Infect diseases. (2010) 10:969–75. doi: 10.1016/j.meegid.2010.06.006 20601170

[116] Asadpour A Riazi-Rad F Khaze V Ajdary S Alimohammadian MH. Distinct strains of Leishmania major induce different cytokine mRNA expression in draining lymph node of BALB/c mice. Parasite Immunol. (2013) 35:42–50. doi: 10.1111/pim.2012.35.issue-1 23106526

[117] Darabi S Khaze V Riazi-Rad F Darabi H Bahrami F Ajdary S. Leishmania major strains isolated from distinct endemic areas show diverse cytokine mRNA expression levels in C57BL/6 mice: Toward selecting an ideal strain for the vaccine studies. Cytokine. (2015) 76:303–8. doi: 10.1016/j.cyto.2015.05.022 26072430

[118] Hosseini M Nateghi Rostami M Hosseini Doust R Khamesipour A. Multilocus sequence typing analysis of Leishmania clinical isolates from cutaneous leishmaniasis patients of Iran. Infection Genet evolution: J Mol Epidemiol evolutionary Genet Infect diseases. (2020) 85:104533. doi: 10.1016/j.meegid.2020.104533 32919066

[119] Uzonna JE Wei G Yurkowski D Bretscher P. Immune elimination of Leishmania major in mice: implications for immune memory, vaccination, and reactivation disease. J Immunol. (2001) 167:6967–74. doi: 10.4049/jimmunol.167.12.6967 11739516

[120] Lira R Doherty M Modi G Sacks D. Evolution of lesion formation, parasitic load, immune response, and reservoir potential in C57BL/6 mice following high- and low-dose challenge with Leishmania major. Infect Immun. (2000) 68:5176–82. doi: 10.1128/IAI.68.9.5176-5182.2000 PMC101773 10948141

[121] Belkaid Y Mendez S Lira R Kadambi N Milon G Sacks D. A natural model of Leishmania major infection reveals a prolonged “silent” phase of parasite amplification in the skin before the onset of lesion formation and immunity. J Immunol. (2000) 165:969–77. doi: 10.4049/jimmunol.165.2.969 10878373

[122] Loeuillet C Banuls AL Hide M. Study of Leishmania pathogenesis in mice: experimental considerations. Parasit Vectors. (2016) 9:144. doi: 10.1186/s13071-016-1413-9 26969511 PMC4788862

[123] Ji J Sun J Qi H Soong L. Analysis of T helper cell responses during infection with Leishmania amazonensis. Am J Trop Med hygiene. (2002) 66:338–45. doi: 10.4269/ajtmh.2002.66.338 12164286

[124] Soong L Chang CH Sun J Longley BJ Jr. Ruddle NH Flavell RA. Role of CD4+ T cells in pathogenesis associated with Leishmania amazonensis infection. J Immunol. (1997) 158:5374–83. doi: 10.4049/jimmunol.158.11.5374 9164958

[125] Afonso LC Scott P. Immune responses associated with susceptibility of C57BL/10 mice to Leishmania amazonensis. Infect Immun. (1993) 61:2952–9. doi: 10.1128/iai.61.7.2952-2959.1993 PMC280944 8514400

[126] Childs GE Lightner LK McKinney L Groves MG Price EE Hendricks LD. Inbred mice as model hosts for cutaneous leishmaniasis. I. Resistance and susceptibility to infection with Leishmania Braziliensis, L. mexicana and L. aethiopica. Ann Trop Med Parasitol. (1984) 78:25–34. doi: 10.1080/00034983.1984.11811769 6721612

[127] Pereira CG Silva AL de Castilhos P Mastrantonio EC Souza RA Romao RP. Different isolates from Leishmania Braziliensis complex induce distinct histopathological features in a murine model of infection. Veterinary Parasitol. (2009) 165:231–40. doi: 10.1016/j.vetpar.2009.07.019 19656631

[128] DeKrey GK Lima HC Titus RG. Analysis of the immune responses of mice to infection with Leishmania Braziliensis. Infect Immun. (1998) 66:827–9. doi: 10.1128/IAI.66.2.827-829.1998 PMC107977 9453649

[129] de Moura TR Novais FO Oliveira F Clarencio J Noronha A Barral A. Toward a novel experimental model of infection to study American cutaneous leishmaniasis caused by Leishmania Braziliensis. Infect Immun. (2005) 73:5827–34. doi: 10.1128/IAI.73.9.5827-5834.2005 PMC1231065 16113301

[130] Oliveira DM Costa MA Chavez-Fumagalli MA Valadares DG Duarte MC Costa LE. Evaluation of parasitological and immunological parameters of Leishmania chagasi infection in BALB/c mice using different doses and routes of inoculation of parasites. Parasitol Res. (2012) 110:1277–85. doi: 10.1007/s00436-011-2628-5 21915627

[131] Honore S Garin YJ Sulahian A Gangneux JP Derouin F. Influence of the host and parasite strain in a mouse model of visceral Leishmania infantum infection. FEMS Immunol Med Microbiol. (1998) 21:231–9. doi: 10.1016/S0928-8244(98)00079-0 9718213

[132] Ahmed S Colmenares M Soong L Goldsmith-Pestana K Munstermann L Molina R. Intradermal infection model for pathogenesis and vaccine studies of murine visceral leishmaniasis. Infect Immun. (2003) 71:401–10. doi: 10.1128/IAI.71.1.401-410.2003 PMC143149 12496190

[133] Kaye PM Curry AJ Blackwell JM. Differential production of Th1- and Th2-derived cytokines does not determine the genetically controlled or vaccine-induced rate of cure in murine visceral leishmaniasis. J Immunol. (1991) 146:2763–70. doi: 10.4049/jimmunol.146.8.2763 1901883

[134] Murray HW. Endogenous interleukin-12 regulates acquired resistance in experimental visceral leishmaniasis. J Infect diseases. (1997) 175:1477–9. doi: 10.1086/jid.1997.175.issue-6 9180189

[135] Murray HW Hariprashad J Coffman RL. Behavior of visceral Leishmania donovani in an experimentally induced T helper cell 2 (Th2)-associated response model. J Exp Med. (1997) 185:867–74. doi: 10.1084/jem.185.5.867 PMC2196164 9120392

[136] Murray HW. Tissue granuloma structure-function in experimental visceral leishmaniasis. Int J Exp pathology. (2001) 82:249–67. doi: 10.1046/j.1365-2613.2001.00199.x PMC2517779 11703536

[137] Neal RA Hale C. A comparative study of susceptibility of inbred and outbred mouse strains compared with hamsters to infection with New World cutaneous leishmaniases. Parasitology. (1983) 87:7–13. doi: 10.1017/S0031182000052379 6622066

[138] Munoz-Durango N Gomez A Garcia-Valencia N Roldan M Ochoa M Bautista-Erazo DE. A mouse model of ulcerative cutaneous leishmaniasis by leishmania (Viannia) panamensis to investigate infection, pathogenesis, immunity, and therapeutics. Front Microbiol. (2022) 13:907631. doi: 10.3389/fmicb.2022.907631 35770175 PMC9234518

[139] Nabors GS Farrell JP. Site-specific immunity to Leishmania major in SWR mice: the site of infection influences susceptibility and expression of the antileishmanial immune response. Infect Immun. (1994) 62:3655–62. doi: 10.1128/iai.62.9.3655-3662.1994 PMC303015 8063382

[140] Krishnan L Guilbert LJ Wegmann TG Belosevic M Mosmann TR. T helper 1 response against Leishmania major in pregnant C57BL/6 mice increases implantation failure and fetal resorptions. Correlation with increased IFN-gamma and TNF and reduced IL-10 production by placental cells. J Immunol. (1996) 156:653–62. doi: 10.4049/jimmunol.156.2.653 8543817

[141] Satoskar AR Rodig S Telford SR 3rd Satoskar AA Ghosh SK von Lichtenberg F. IL-12 gene-deficient C57BL/6 mice are susceptible to Leishmania donovani but have diminished hepatic immunopathology. Eur J Immunol. (2000) 30:834–9. doi: 10.1002/1521-4141(200003)30:3<834::AID-IMMU834>3.0.CO;2-9 10741399

[142] Murray HW Hariprashad J. Interleukin 12 is effective treatment for an established systemic intracellular infection: experimental visceral leishmaniasis. J Exp Med. (1995) 181:387–91. doi: 10.1084/jem.181.1.387 PMC2191839 7807019

[143] Engwerda CR Kaye PM. Organ-specific immune responses associated with infectious disease. Immunol Today. (2000) 21:73–8. doi: 10.1016/S0167-5699(99)01549-2 10652464

[144] Melby PC Tabares A Restrepo BI Cardona AE McGuff HS Teale JM. Leishmania donovani: evolution and architecture of the splenic cellular immune response related to control of infection. Exp Parasitol. (2001) 99:17–25. doi: 10.1006/expr.2001.4640 11708830

[145] Rodrigues OR Marques C Soares-Clemente M Ferronha MH Santos-Gomes GM. Identification of regulatory T cells during experimental Leishmania infantum infection. Immunobiology. (2009) 214:101–11. doi: 10.1016/j.imbio.2008.07.001 19167988

[146] Squires KE Kirsch M Silverstein SC Acosta A McElrath MJ Murray HW. Defect in the tissue cellular immune response: experimental visceral leishmaniasis in euthymic C57BL/6 ep/ep mice. Infect Immun. (1990) 58:3893–8. doi: 10.1128/iai.58.12.3893-3898.1990 PMC313751 2123825

[147] Baptista-Fernandes T Marques C Roos Rodrigues O Santos-Gomes GM. Intra-specific variability of virulence in Leishmania infantum zymodeme MON-1 strains. Comp immunology Microbiol Infect diseases. (2007) 30:41–53. doi: 10.1016/j.cimid.2006.10.001 17109961

[148] Ferreira-Paes T Charret KDS Ribeiro M Rodrigues RF Leon LL. Comparative analysis of biological aspects of Leishmania infantum strains. PloS One. (2020) 15:e0230545. doi: 10.1371/journal.pone.0230545 33270636 PMC7714135

[149] Kaur S Kaur T Garg N Mukherjee S Raina P Athokpam V. Effect of dose and route of inoculation on the generation of CD4+ Th1/Th2 type of immune response in murine visceral leishmaniasis. Parasitol Res. (2008) 103:1413–9. doi: 10.1007/s00436-008-1150-x 18751727

[150] Rolao N Melo C Campino L. Influence of the inoculation route in BALB/c mice infected by Leishmania infantum. Acta tropica. (2004) 90:123–6. doi: 10.1016/j.actatropica.2003.09.010 14739031

[151] Rosypal AC Zajac AM Troy GC Lindsay DS. Infections in immunocompetent and immune-deficient mice with promastigotes of a North American isolate of Leishmania infantum. Veterinary Parasitol. (2005) 130:19–27. doi: 10.1016/j.vetpar.2005.03.017 15893066

[152] Requena JM Soto M Doria MD Alonso C. Immune and clinical parameters associated with Leishmania infantum infection in the golden hamster model. Veterinary Immunol immunopathology. (2000) 76:269–81. doi: 10.1016/S0165-2427(00)00221-X 11044559

[153] Melby PC Chandrasekar B Zhao W Coe JE. The hamster as a model of human visceral leishmaniasis: progressive disease and impaired generation of nitric oxide in the face of a prominent Th1-like cytokine response. J Immunol. (2001) 166:1912–20. doi: 10.4049/jimmunol.166.3.1912 11160239

[154] Nieto A Dominguez-Bernal G Orden JA de la Fuente R Madrid-Elena N Carrion J. Mechanisms of resistance and susceptibility to experimental visceral leishmaniosis: BALB/c mouse versus Syrian hamster model. Veterinary Res. (2011) 42:39. doi: 10.1186/1297-9716-42-39 PMC3052183 21345200

[155] Mukherjee P Ghosh AK Ghose AC. Infection pattern and immune response in the spleen and liver of BALB/c mice intracardially infected with Leishmania donovani amastigotes. Immunol letters. (2003) 86:131–8. doi: 10.1016/S0165-2478(03)00021-X 12644314

[156] Carrion J Nieto A Iborra S Iniesta V Soto M Folgueira C. Immunohistological features of visceral leishmaniasis in BALB/c mice. Parasite Immunol. (2006) 28:173–83. doi: 10.1111/j.1365-3024.2006.00817.x 16629702

[157] Alexander J. Sex differences and cross-immunity in DBA/2 mice infected with L. mexicana and L. major. Parasitology. (1988) 96:297–302. doi: 10.1017/S0031182000058303 3374966

[158] Demant P Hart AA. Recombinant congenic strains–a new tool for analyzing genetic traits determined by more than one gene. Immunogenetics. (1986) 24:416–22. doi: 10.1007/BF00377961 3793154

[159] Dutta S Sengupta P. Men and mice: Relating their ages. Life Sci. (2016) 152:244–8. doi: 10.1016/j.lfs.2015.10.025 26596563

[160] Sohrabi Y Reinecke H Joosten LAB Netea MG. Deadly COVID-19 among the elderly: Innate immune memory helping those most in need. Med. (2021) 2:378–83. doi: 10.1016/j.medj.2021.02.004 PMC7903897 33649749

[161] Salgado CL Corea AFM Covre LP Fonseca-Martins AMD Falqueto A Guedes HLM. Intranasal delivery of LaAg vaccine improves immunity of aged mice against visceral Leishmaniasis. Acta tropica. (2024) 252:107125. doi: 10.1016/j.actatropica.2024.107125 38280636

[162] Boy-Waxman S Olivier M Cermakian N. Clockwork intruders: Do parasites manipulate their hosts’ circadian rhythms? Curr Res Parasitol Vector Borne Dis. (2024) 5:100171. doi: 10.1016/j.crpvbd.2024.100171 38545439 PMC10966150

[163] Dominguez-Andres J Reinecke H Sohrabi Y. The immune hunger games: the effects of fasting on monocytes. Cell Mol Immunol. (2023) 20:1098–100. doi: 10.1038/s41423-023-01033-w PMC10541887 37165013

[164] Pick R He W Chen CS Scheiermann C. Time-of-day-dependent trafficking and function of leukocyte subsets. Trends Immunol. (2019) 40:524–37. doi: 10.1016/j.it.2019.03.010 31109762

[165] Zhang Q Schwarz D Cheng Y Sohrabi Y. Unraveling host genetics and microbiome genome crosstalk: a novel therapeutic approach. Trends Mol Med. (2024) 30:1007–9. doi: 10.1016/j.molmed.2024.06.007 38937208

[166] Misra P Singh S. Site specific microbiome of Leishmania parasite and its cross-talk with immune milieu. Immunol letters. (2019) 216:79–88. doi: 10.1016/j.imlet.2019.10.004 31678358

[167] Mrazek J Mrazkova L Mekadim C Jarosikova T Krayem I Sohrabi Y. Effects of Leishmania major infection on the gut microbiome of resistant and susceptible mice. Appl Microbiol Biotechnol. (2024) 108:145. doi: 10.1007/s00253-024-13002-y 38240984 PMC10799115

[168] Meazzi S Lauzi S Martini V Ferriani R Peri M Zanzani SA. Gut microbiota and lymphocyte subsets in canine leishmaniasis. Front Vet Sci. (2022) 9:868967. doi: 10.3389/fvets.2022.868967 35909678 PMC9326463

[169] Sarnaglia GD Covre LP Pereira FE HL DEMG Faria AM Dietze R. Diet-induced obesity promotes systemic inflammation and increased susceptibility to murine visceral leishmaniasis. Parasitology. (2016) 143:1647–55. doi: 10.1017/S003118201600127X 27440305

[170] Lago T Carvalho LP Nascimento M Guimaraes LH Lago J Castellucci L. Influence of obesity on clinical manifestations and response to therapy in cutaneous leishmaniasis caused by leishmania Braziliensis. Clin Infect Dis. (2021) 73:1020–6. doi: 10.1093/cid/ciab236 PMC8442773 33725723

[171] Martins VD Vaz L Barbosa SC Paixao PHM Torres L de Oliveira MFA. Obesity alters the macrophages’ response to Leishmania major in C57BL/6 mice. J Leukoc Biol. (2024) 116:1372–84. doi: 10.1093/jleuko/qiae171 39213305

[172] Amorim Sacramento L Gonzalez-Lombana C Scott P. Malnutrition disrupts adaptive immunity during visceral leishmaniasis by enhancing IL-10 production. PloS Pathog. (2024) 20:e1012716. doi: 10.1101/2024.06.06.597776 39527629 PMC11581394

[173] Osorio EY Uscanga-Palomeque A Patterson GT Cordova E Travi BL Soong L. Malnutrition-related parasite dissemination from the skin in visceral leishmaniasis is driven by PGE2-mediated amplification of CCR7-related trafficking of infected inflammatory monocytes. PloS Negl Trop Dis. (2023) 17:e0011040. doi: 10.1371/journal.pntd.0011040 36630476 PMC9873180

[174] Nweze JA Nweze EI Onoja US. Nutrition, malnutrition, and leishmaniasis. Nutrition. (2020) 73:110712. doi: 10.1016/j.nut.2019.110712 32078915

[175] Bennis I De Brouwere V Belrhiti Z Sahibi H Boelaert M. Psychosocial burden of localised cutaneous Leishmaniasis: a scoping review. BMC Public Health. (2018) 18:358. doi: 10.1186/s12889-018-5260-9 29544463 PMC5855994

[176] Grifferty G Shirley H McGloin J Kahn J Orriols A Wamai R. Vulnerabilities to and the socioeconomic and psychosocial impacts of the leishmaniases: A review. Res Rep Trop Med. (2021) 12:135–51. doi: 10.2147/RRTM.S278138 PMC8236266 34188584

[177] Pires M Wright B Kaye PM da Conceicao V Churchill RC. The impact of leishmaniasis on mental health and psychosocial well-being: A systematic review. PloS One. (2019) 14:e0223313. doi: 10.1371/journal.pone.0223313 31622369 PMC6797112

[178] Chahed MK Bellali H Ben Jemaa S Bellaj T. Psychological and psychosocial consequences of zoonotic cutaneous leishmaniasis among women in Tunisia: preliminary findings from an exploratory study. PloS Negl Trop Dis. (2016) 10:e0005090. doi: 10.1371/journal.pntd.0005090 27788184 PMC5082956

[179] Portes A Giestal-de-Araujo E Fagundes A Pandolfo P de Sa Geraldo A Lira ML. Leishmania amazonensis infection induces behavioral alterations and modulates cytokine and neurotrophin production in the murine cerebral cortex. J Neuroimmunol. (2016) 301:65–73. doi: 10.1016/j.jneuroim.2016.11.003 27876366

[180] Pal B Murti K Siddiqui NA Das P Lal CS Babu R. Assessment of quality of life in patients with post kalaazar dermal leishmaniasis. Health Qual Life Outcomes. (2017) 15:148. doi: 10.1186/s12955-017-0720-y 28738881 PMC5525288

[181] Garapati P Pal B Siddiqui NA Bimal S Das P Murti K. Knowledge, stigma, health seeking behaviour and its determinants among patients with post kalaazar dermal leishmaniasis, Bihar, India. PloS One. (2018) 13:e0203407. doi: 10.1371/journal.pone.0203407 30192805 PMC6128567

[182] Amni F Maleki-Ravasan N Nateghi-Rostami M Hadighi R Karimian F Meamar AR. Corrigendum: Co-infection of Phlebotomus papatasi (Diptera: Psychodidae) gut bacteria with Leishmania major exacerbates the pathological responses of BALB/c mice. Front Cell Infect Microbiol. (2023) 13:1185912. doi: 10.3389/fcimb.2023.1185912 37065207 PMC10098325

[183] Rohousova I Volf P Lipoldova M. Modulation of murine cellular immune response and cytokine production by salivary gland lysate of three sand fly species. Parasite Immunol. (2005) 27:469–73. doi: 10.1111/j.1365-3024.2005.00787.x 16255746

[184] Lestinova T Rohousova I Sima M de Oliveira CI Volf P. Insights into the sand fly saliva: Blood-feeding and immune interactions between sand flies, hosts, and Leishmania. PloS Negl Trop Dis. (2017) 11:e0005600. doi: 10.1371/journal.pntd.0005600 28704370 PMC5509103

[185] Abdeladhim M Kamhawi S Valenzuela JG. What’s behind a sand fly bite? The profound effect of sand fly saliva on host hemostasis, inflammation and immunity. Infection Genet evolution: J Mol Epidemiol evolutionary Genet Infect diseases. (2014) 28:691–703. doi: 10.1016/j.meegid.2014.07.028 PMC4562216 25117872

[186] Rogers ME Ilg T Nikolaev AV Ferguson MA Bates PA. Transmission of cutaneous leishmaniasis by sand flies is enhanced by regurgitation of fPPG. Nature. (2004) 430:463–7. doi: 10.1038/nature02675 PMC2835460 15269771

[187] Hessler JR. The history of environmental improvements in laboratory animal science: caging, systems, equipment, and facility design. Fifty Years of Laboratory Animal Science. Memphis, TN: American Association for Laboratory Animal Science (1999).

[188] Foster HL. Housing of disease-free vertebrates. Ann N Y Acad Sci. (1959) 78:80–8. doi: 10.1111/j.1749-6632.1959.tb53096.x 13824112

[189] Dobson GP Letson HL Biros E Morris J. Specific pathogen-free (SPF) animal status as a variable in biomedical research: Have we come full circle? EBioMedicine. (2019) 41:42–3. doi: 10.1016/j.ebiom.2019.02.038 PMC6443024 30803932

[190] Beura LK Hamilton SE Bi K Schenkel JM Odumade OA Casey KA. Normalizing the environment recapitulates adult human immune traits in laboratory mice. Nature. (2016) 532:512–6. doi: 10.1038/nature17655 PMC4871315 27096360

[191] Karimkhani C Wanga V Coffeng LE Naghavi P Dellavalle RP Naghavi M. Global burden of cutaneous leishmaniasis: a cross-sectional analysis from the Global Burden of Disease Study 2013. Lancet Infect Dis. (2016) 16:584–91. doi: 10.1016/S1473-3099(16)00003-7 26879176

[192] Pigott DM Bhatt S Golding N Duda KA Battle KE Brady OJ. Global distribution maps of the leishmaniases. Elife. (2014) 3:e02851. doi: 10.7554/eLife.02851 24972829 PMC4103681

[193] Alvar J Velez ID Bern C Herrero M Desjeux P Cano J. Leishmaniasis worldwide and global estimates of its incidence. PloS One. (2012) 7:e35671. doi: 10.1371/journal.pone.0035671 22693548 PMC3365071

[194] Knight CA Harris DR Alshammari SO Gugssa A Young T Lee CM. Leishmaniasis: Recent epidemiological studies in the Middle East. Front Microbiol. (2022) 13:1052478. doi: 10.3389/fmicb.2022.1052478 36817103 PMC9932337

[195] van Henten S Adriaensen W Fikre H Akuffo H Diro E Hailu A. Cutaneous leishmaniasis due to leishmania aethiopica. EClinicalMedicine. (2018) 6:69–81. doi: 10.1016/j.eclinm.2018.12.009 31193672 PMC6537575

[196] de Freitas Milagres T Lopez-de-Felipe M da Silva WJ Martin-Martin I Galvez R da Silva OS. Same parasite, different outcomes: unraveling the epidemiology of Leishmania infantum infection in Brazil and Spain. Trends Parasitol. (2023) 39:774–85. doi: 10.1016/j.pt.2023.06.008 37442747

[197] Herrera G Barragan N Luna N Martinez D De Martino F Medina J. An interactive database of Leishmania species distribution in the Americas. Sci Data. (2020) 7:110. doi: 10.1038/s41597-020-0451-5 32245983 PMC7125201

[198] Gramiccia M Gradoni L. The current status of zoonotic leishmaniases and approaches to disease control. Int J Parasitol. (2005) 35:1169–80. doi: 10.1016/j.ijpara.2005.07.001 16162348

[199] Soares L Abad-Franch F Ferraz G. Epidemiology of cutaneous leishmaniasis in central Amazonia: a comparison of sex-biased incidence among rural settlers and field biologists. Trop Med Int Health. (2014) 19:988–95. doi: 10.1111/tmi.2014.19.issue-8 24862350

[200] Doherty TM Coffman RL. Leishmania major: effect of infectious dose on T cell subset development in BALB/c mice. Exp Parasitol. (1996) 84:124–35. doi: 10.1006/expr.1996.0098 8932762

[201] Uzonna JE Joyce KL Scott P. Low dose Leishmania major promotes a transient T helper cell type 2 response that is down-regulated by interferon gamma-producing CD8+ T cells. J Exp Med. (2004) 199:1559–66. doi: 10.1084/jem.20040172 PMC2211781 15184505

[202] Bretscher PA Wei G Menon JN Bielefeldt-Ohmann H. Establishment of stable, cell-mediated immunity that makes “susceptible” mice resistant to Leishmania major. Science. (1992) 257:539–42. doi: 10.1126/science.1636090 1636090

[203] Anderson CF Lira R Kamhawi S Belkaid Y Wynn TA Sacks D. IL-10 and TGF-β Control the establishment of persistent and transmissible infections produced by leishmania tropica in C57BL/6 mice. J Immunol. (2008) 180:4090–7. doi: 10.4049/jimmunol.180.6.4090 18322219

